# Integrative analysis of proteomics and metabolomics reveals amino acid metabolism disorder in adriamycin-resistant acute myeloid leukemia cells

**DOI:** 10.1038/s41598-026-35675-3

**Published:** 2026-01-09

**Authors:** Cong Li, Xue Liang, Siqi Gong, Mengmeng Fan, Yanghua Tian, Qiang Hong, Zhimin Zhai

**Affiliations:** 1 Department of Hematology, The Second Affiliated Hospital of Anhui Medical University, Hefei, 230601 China; 2 School of Basic Medical Sciences, Anhui Medical University, Hefei, 230032 China; 3 Department of Neurology, The Second Affiliated Hospital of Anhui Medical University, Hefei, 230601 China

**Keywords:** Proteomics, Metabolomics, Acute myeloid leukemia, Adriamycin, Drug resistance, Leukaemia, Acute myeloid leukaemia

## Abstract

Acute myeloid leukemia (AML) is a complex and aggressive hematopoietic malignancy. Clinically, adriamycin is an important therapeutic drug. However, drug resistance often leads to refractory or relapse of patients after anthracycline-based treatment, and the prognosis is poor. Despite extensive research, the molecular mechanisms underlying adriamycin resistance in AML remain elusive. 4D label-free quantitative proteomics and untargeted metabolomics techniques were used to quantify and analyze the protein and metabolic profiles of HL60 and adriamycin-resistant cell line HL60/R. A total of 3,241 proteins were differentially expressed, including 1,686 up-regulated and 1,555 down-regulated proteins, and 260 metabolites were differentially expressed with 79 up-regulated and 41 down-regulated under positive ion mode, and 77 up-regulated and 63 down-regulated under negative ion mode in HL60/R compared to HL60. Subsequently, bioinformatics analysis of the altered proteins showed that they were involved in many biological processes, particularly cAMP signaling pathway, HIF-1 signaling pathway and oxidative phosphorylation. Furthermore, an integrated analysis of proteome and metabolome showed that the significantly altered metabolism pathways were mainly involved in amino acid metabolism, such as alanine, aspartate and glutamate metabolism, cysteine and methionine metabolism and glutathione metabolism, which indicated that they may participated in the pathogenesis of drug resistance in HL60 cells. Finally, six significantly changed proteins including GOT1, GPX1, AHCY, MAT2A, BCAT1, GCLM in amino acid metabolism were further validated through western blot. MAT2A, BCAT1 and GCLM were selected for small interfering RNA experiments and the results exhibited that apoptosis rates significantly promoted in HL60/R when MAT2A, BCAT1 or GCLM were interfered. An integrative analysis of proteomic and metabolomic data revealed significant disruptions in amino acid metabolism in HL60/R cells, including the metabolic pathways of alanine, aspartate and glutamate, cysteine and methionine, as well as glutathione metabolism.

**Supplementary Information:**

The online version contains supplementary material available at 10.1038/s41598-026-35675-3.

## Introduction

Acute myeloid leukemia (AML) is a complicated, highly heterogeneous and aggressive hematopoietic malignancy characterized by abnormal cell proliferation and differentiation of malignant clonal population of myeloid stem cells and myeloblasts^1^. The clinical manifestations of AML mainly include various forms of pancytopenia, as well as constitutional symptoms^2^. Epidemiological studies have shown that AML is the most common acute leukemia, and it can affect people of all ages, especially in elderly individuals (older than 65 years)^3^. Clinically, eligible patients are first treated with induction chemotherapy, such as anthracycline-based therapy, followed by allogeneic stem cell transplantation or additional chemotherapy during a consolidation phase to achieve complete remission (CR). Unfortunately, some patients have a refractory disease, and the majority of patients will experience a relapse after they have achieved CR^4,5^. Although several novel agents, such as FLT3 (FMS-related receptor tyrosine kinase 3)-ITD (internal tandem duplication) inhibitors, STAT (Signal Transducer and Activator of Transcription proteins) inhibitors, IDH1/IDH2 ( isocitrate dehydrogenase 1/2) small molecule inhibitors, clofarabine, monoclonal antibodies (Gemtuzumab ozogamicin and AGS67E) and CAR-T (chimeric antigen receptor-modified T cells) therapy have promoted the treatment efficacy of AML, the prognosis in the elderly population remains poor^6^. Of note, previous literature has indicated that metabolic perturbations and cellular reprogramming play important roles in leukemogenesis and disease progression^7^. However, the underlying regulatory relationships remain elusive. Therefore, it is critical to identify and control leukemia-specific metabolic vulnerabilities, which will enhance our understanding of the pathogenesis of AML and can provide new potential targets for AML therapy^8^.

In the past few decades, great progress has been made in AML treatment due to the emergence of several new therapies. However, with the use of new therapeutic drugs, side effects and drug resistance are also manifested in patients^9^. Anthracycline-based chemotherapeutic agents, such as adriamycin and daunorubicin, remain the treatment of choice in the clinical treatment of AML patients. Cytotoxic chemotherapy, with or without targeted therapy, remains the gold standard for patients with AML^10^. Nevertheless, the effects of chemotherapy are often limited due to the occurrence of drug resistance. Thus, it is imperative to explore the underlying mechanism of drug resistance in AML. In fact, resistance to anti-tumor drugs may be either acquired (caused by drug treatment) or intrinsic (pre-existing and not caused by the drug)^11^. Multiple factors are involved in the development of drug resistance, which include tumor factors, host factors, and tumor-host interactions. Interestingly, an outward transport mechanism exists in P388 leukemia cells, and when anthracyclines are administered, the enhanced activity of this efflux process renders the cells highly resistant to the cytotoxic effects of anthracyclines^12^.Several ATP (adenosine triphosphate) -binding cassette (ABC) transporters were involved in the active drug efflux system, such as P-glycoprotein (P-gp) and MRP1 (multidrug resistance-associated protein)^13–15^. In addition to that, activation of compensatory pathways, deregulation of cell death pathways, enhancement of DNA (deoxyribonucleic acid) repair mechanisms, mutation of drug targets, and deregulation of metabolic pathways are also implicated in drug resistance^16^. Although various mechanisms associated with drug resistance have been widely reported, adriamycin resistance is still ongoing. Thus, the pivotal regulatory factors of adriamycin resistance need to be further investigated.

Transcriptomics, proteomics, and metabolomics technologies have been utilized to identify potential therapeutic targets in acute myeloid leukemia^17–19^. Nevertheless, there is a dearth of joint analysis of proteomics and metabolomics data for drug-sensitive and drug-resistant cells in AML. Integrating multi-omics data analysis can compensate for data noise, loss and other factors in single omic data analysis, which can elucidate the overall state of biological system. The combined application of metabolomics and proteomics has great advantages^20,21^. Thus, it is important to identify novel targets and key metabolic pathways that are associated with drug resistance by integrating proteomics with untargeted metabolomics. In the present study, proteomic and metabolomic techniques were used to quantify and analyze the protein and metabolic profiles of HL60 and adriamycin-resistant cell line HL60/R. Integrative analysis revealed significant alterations in amino acid metabolism in HL60/R cells. These findings provide valuable resources for further study on the mechanism of adriamycin resistance in acute myeloid leukemia cells.

## Materials and methods

### Cell culture and adriamycin treatment

The human AML cell line HL60 was purchased from Shanghai Cell Bank (Shanghai, China). The adriamycin-resistant cell line HL60/R was generously provided by the Central Laboratory of the First Hospital of Lanzhou University^22^. The adriamycin-resistance of HL60/R was validated by IC50 (Inhibitory concentration 50, half maximal inhibitory concentration) and apoptosis assay (Fig. S1). HL60 cells were routinely cultured in RPMI-1640 media with 10% FBS (foetal bovine serum) and penicillin/streptomycin (1%) in a 5% CO_2_ and 37 °C cell incubator. For HL60/R cells, the culture conditions were similar to HL60 cells, except that adriamycin with a final concentration of 1 µg/ml was added to the medium. The cells were maintained in adriamycin-free medium for one week, and adriamycin was eliminated before subsequent experiments.

### Proteomics analysis

In this study, 4D label-free proteomics was performed to qualitative and quantitative analysis of differential proteins. Primary experimental procedures included protein preparation, liquid crystal tandem mass spectrometry (LC-MS/MS) analysis, and bioinformatic analysis. Shortly, the proteins were extracted from HL60 (*n* = 3) and HL60/R (*n* = 3) cells. Each group of cells was collected and suspended in a frozen PBS, and then the samples were homogenized in lysis buffer (4% SDS, 150 mM Tris-HCl pH 8.0, 1mM DTT and 1% protease inhibitor) for 30 min, followed by sonication on ice. After centrifugation, the concentrations of proteins were quantified using the BCA Protein Assay Kit (P0010, Beyotime). Subsequently, 20 µg protein of each sample was separated with SDS-PAGE to assess protein quality. Each protein sample was mixed and digested with trypsin based on the filter-aided sample preparation (FASP) procedure, and then LC-MS/MS was performed using a Nanoelute UHPLC (ultra high pressure liquid chromatography) system coupled with a timsTOF-Pro mass spectrometer (Bruker, Germany) in Applied Protein Technology (Shanghai, China). The obtained peptides were loaded onto a reverse-phase trap column (Thermo Scientific Acclaim PepMap100, 100 μm * 2 cm, nanoViper C18) that connected to the C18-reversed-phase analytical column (Thermo Scientific Easy Column) in buffer A (0.1% Formic acid) and separated by a linear gradient of buffer B (0.1% Formic acid and 84% acetonitrile) under a flow rate of 300 nl/min, governed by IntelliFlow technology. The mass spectrometer was operated under positive ion mode. The mass spectrometer collected an ion mobility mass spectrum with a mass range of m/z 100–1700 and a mass range of 1/k0 0.6–1.6 with a target intensity of 1.5k and a threshold of 2500 for 10 parallel acumulative serial fragmentation (PASEF) MS/MS cycles. The secondary spectrum of the charges in the 0–5 range is actively excluded and the release time is 0.4 min. The raw MS/MS data was collected and searched with MaxQuant 1.5.3.17 software for quantitation analysis. The label free quantification (LFQ), which was provided by the MaxQuant software, was used for protein quantification between groups. And the main algorithm is paired correction using multiple layers of peptides and proteins.

### Screening of differential proteins and bioinformatic analysis

For the differentially expressed proteins (DEPs) analysis, the proteins that with a fold change (FC) > 2 or < 0.5 and adjusted p -values below 0.05 (FDR < 0.05) were were defined as significant up-regulated or down-regulated differently expressed proteins. Subsequently, bioinformatic pathway analysis was performed. The selected differentially expressed protein sequences were locally searched by NCBI BLAST + client software and InterProScan was used to find homologous sequences. After that, Blast2GO software was used to map the terms of gene ontology (GO) and annotate the sequences. The GO annotation included cellular component (CC), biological process (BP), and molecular function (MF), and the results were visualized by R software package. Simultaneously, the obtained DEPs were blasted in the Kyoto Encyclopedia of Genes and Genomes (KEGG) public database for retrieving KEGG identifications and then mapped to the KEGG database pathways. Enrichment analysis was conducted according to Fisher’s exact test, with the whole quantitative protein as background dataset. Furthermore, the Benjamini-Hochberg correction of the multivariate test was used to adjust the p-value. Specifically, pathways and functional categories with a p-value < 0.05 were deemed to be significant. For protein-protein interaction (PPI) analysis, the PPI information of the proteins was obtained through its gene symbol or a STRING software in IntAct molecular interaction database. The results were visualized by Cytoscape software.

### Untargeted metabolomics methods

The cells for untargeted metabolomics experiments were prepared as previously described^23^. Samples were stored at -80 °C, prepared on dry ice to minimize metabolic activity, and metabolites were extracted using a methanol–acetonitrile–water mixture to ensure effective metabolic quenching prior to analysis. After centrifugation, concentration, and resuspension, the extracted samples were analyzed by a UHPLC system (1290, Agilent Technologies) coupled with a quadrupole time-of-flight instrument (ABI SCIEX Triple TOF 6600). The quality control samples were used to monitor the repeatability and stability of the instrument analysis. The components of each sample were separated on a Waters ACQUIY UPLC BEH column (1.7 μm, 2.1 mm × 100.0 mm, ) at a temperature of 25 °C. The mobile phase of both ESI positive and negative modes consisted of (A) 25.0 mM ammonium hydroxide/ammonium acetate in water and (B) acetonitrile. All samples are stored at 4 °C during analysis. The injection volume was fixed at 5 µL with the flow rate was 0.4 mL/min, and the linear gradient elution program was following the standard protocols. Next, the QTOF (quadrupole time-of-flight) mass spectrometer was operated by assembling an electrospray ionization (ESI) source. For untargeted metabolomics studies, the instrument is set to obtain automatic MS/MS acquisition of 25-1000 Da in the m/z range, and the product ion scan accumulation time is set to 0.05 s/ spectra. The information dependent acquisition (IDA) with high sensitivity mode selected was used to obtain product ion scan. The parameters: collision energy (CE), 35 ± 15 eV; declustering potential (DP), ± 60 V (± ESI); isotopes up to 4 Da were excluded and 10 candidate ions were monitored per cycle.

### Screening of differentially expressed metabolites (DEMs)

Prior to analysis, the raw data quality was assessed. After sum-normalization, the metabolomic data were used for multivariate statistical analysis, which included principal component analysis (PCA) and partial least-squares discrimination analysis (PLS-DA). Subsequently, metabolites with a variable importance in projection (VIP) > 1 and p-value < 0.05 (student’s t-test) were identified as significant DEMs. Then, pathway enrichment analysis and cluster analysis of DEMs were performed based on MetaboAnalyst 6.0 and Kyoto Encyclopedia of Genes and Genomes (KEGG) database, and p-value < 0.05 was considered significant. Finally, we also analyzed the DEMs using differential abundance score analysis.

### Integration analysis of differential proteins and metabolites

For the integration analysis of proteome and metabolome, the differential proteins and metabolites of this study were selected and mapped to the KEGG database, respectively. The correlation coefficient between differential proteins and metabolites was calculated by Pearson correlation analysis. After that, the relationships between proteome and metabolome were conducted using the Cytoscape software (version 3.4.0).

### Western blot analysis

Western blots were performed as previously described^24^. Simply, the cultured cells were collected and lysed in RIPA buffer supplemented with 1% PMSF (phenylmethylsulfonyl fluoride). After centrifugation, the concentrations of proteins were quantified using the BCA Protein Assay Kit (P0010, Beyotime). The protein extracts were separated by 10% SDS-PAGE and then electro-transferred to polyvinylidene difluoride (PVDF) membranes. After blocking with 5% non-fat milk powder in TBST for 1 h, the membranes were incubated with primary antibodies against GAPDH (glyceraldehyde 3-phosphate dehydrogenase, 1:2000, AC001, ABclonal), GOT1 (glutamic-oxaloacetic transaminase 1, 1:1000, ab221939, Abcam), GPX1 (Glutathione peroxidase 1, 1:1000, ab22604, Abcam), AHCY (S-adenosyl-homocysteine-hydrolase, 1:2000, ab134966, Abcam), MAT2A (methionine adenosyltransferase 2alpha, 1:1000, ab154343, Abcam), BCAT1 (branched-chain amino acid transaminase 1, 1:1000, ab197941, Abcam) and GCLM (glutamate-cysteine ligase modifier subunit, 1:500, ab154017, Abcam) at 4 °C overnight. Following that, the membranes were washed by TBST and incubated with matched secondary antibody conjugated with HRP for 1 h at room temperature. Finally, bands of the membranes were visualized by an ECL detection kit (Beyotime, China) using a gel imaging detector (Bio-Rad, USA). ImageJ software was applied for protein densitometry.

### Small interfering RNAs (siRNAs) transfection

Small interfering RNAs (siRNAs) were synthesized by Tsingke Biotechnology. Lipofectamine 3000 (Invitrogen, USA) were ultilized to transfecting siRNAs for 48 h. The detailed procedures have been previously published^23^. The siRNAs used in the study were as follows:

si-*MAT2A* sense: 5’-CUCAAGUUACUGUGCAGUA-3’.

si-*MAT2A* anti-sense: 5’-UACUGCACAGUAACUUGAG-3’.

si-*BCAT1* sense: 5’-GAACUGUGUUCACGGAUCA-3’.

si-*BCAT1* anti-sense: 5’-UGAUCCGUGAACACAGUUC-3’.

si-*GCLM* sense: 5’-CCAAGAAGCUCUUCAGGAA-3’.

si-*GCLM* anti-sense: 5’-UUCCUGAAGAGCUUCUUGG-3’.

### RNA isolation, cDNA synthesis and qRT-PCR

The total RNA extraction, cDNA synthesis and qRT-PCR refered to the previously study^25^. Primers used in the study were as follows:

*GAPDH* sense: 5’-AGCAAGAGCACAAGAGGAAG-3’.

*GAPDH* anti-sense: 5’-GGTTGAGCACAGGGTACTTT-3’.

*MAT2A* sense: 5’-ATGAACGGACAGCTCAACGG-3’.

*MAT2A* anti-sense: 5’-CCAGCAAGAAGGATCATTCCAG-3’.

*BCAT1* sense: 5’-GTGGAGTGGTCCTCAGAGTTT-3’.

*BCAT1* anti-sense: 5’-AGCCAGGGTGCAATGACAG-3’.

*GCLM* sense: 5’-TGTCTTGGAATGCACTGTATCTC-3’.

*GCLM* anti-sense: 5’-CCCAGTAAGGCTGTAAATGCTC-3’.

### Flow cytometry

Apoptosis was quantified with an Annexin V-APC/PI Apoptosis Detection Kit (BB-41033-100T, BestBio, China) following the manufacturer’s protocol. Harvested cells underwent three washes with phosphate-buffered saline (PBS) before supernatant removal. The pellets were resuspended in binding buffer, then treated with 5 µL Annexin V-APC and 5 µL propidium iodide (PI). Samples were incubated for 15 min at room temperature under light-protected conditions. Flow cytometry analysis (NAVIOS Flow cytometer; Beckman Coulter, USA) measured apoptotic populations, with FlowJo v10.8.1 software processing the resulting data.

### Statistical analysis

The data were performed at least three times, and the values were presented as means ± SEM. Student’s t-test was used for two group statistical comparison. One-way and two-way ANOVA were performed to detect the significance of multiple comparisons. Statistical analysis was performed using GraphPad Prism 6 software. The statistically significant differences were considered when p value < 0.05.

## Results

### Proteomics data analysis

**Fig. 1:**
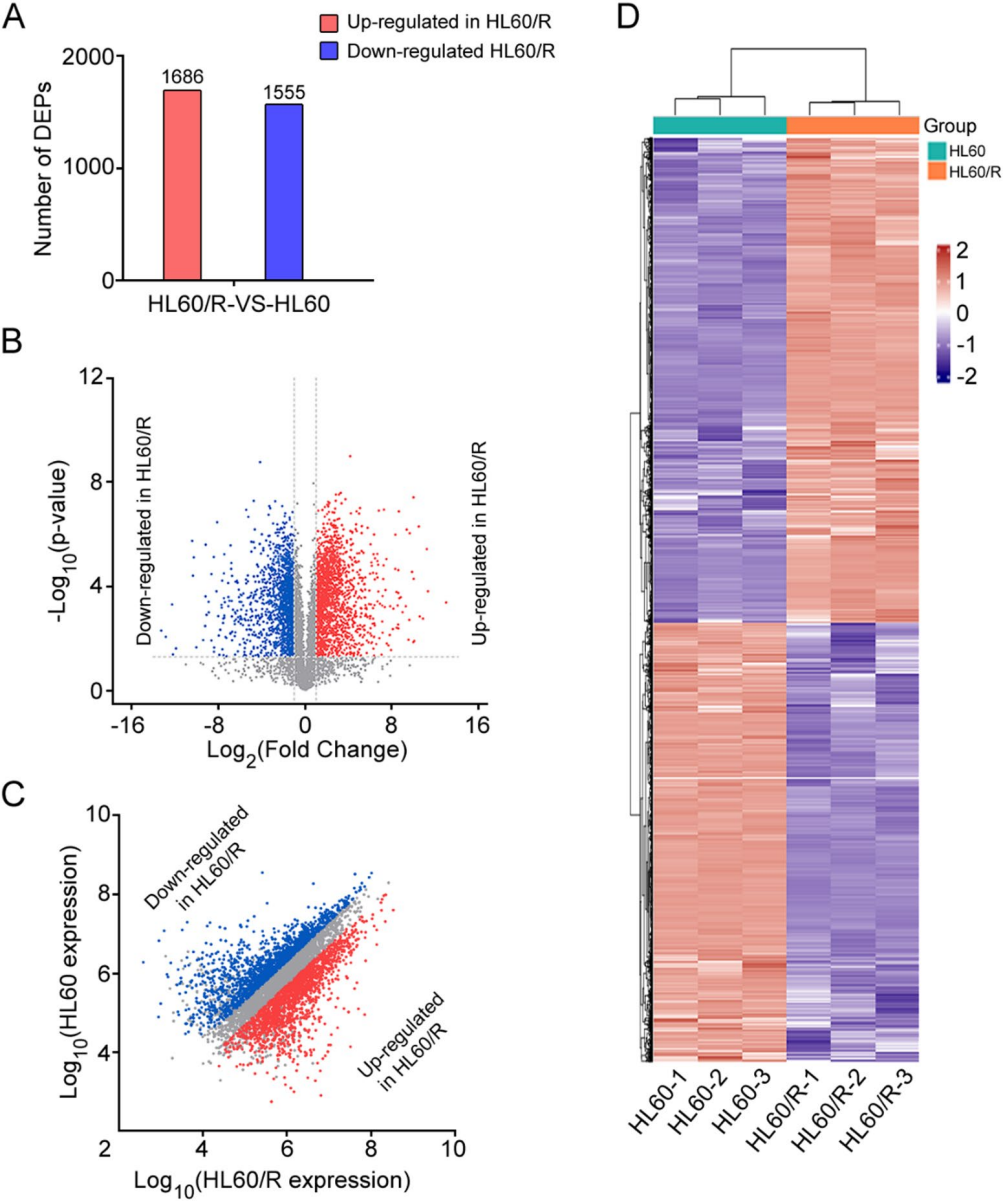
Identification of DEPs between HL60 and HL60/R cells. (**A**) The number of up-regulated and down-regulated DEPs of HL60/R in comparison with HL60 cell. (**B**) Volcano plot indicated the DEPs. The red and blue points represented significantly up-regulated and down-regulated proteins, respectively, while the gray points represented equally expressed proteins. (**C**) Scatter plot showed the DEPs. (**D**) Hierarchical clustering indicated the DEPs and their expression levels.

To explore the potential mechanism of drug resistance induced by adriamycin in HL60 cells, we performed 4D label-free quantitative proteomics analysis of HL60 and its adriamycin-resistant cell line HL60/R to obtain global expression of the protein profiles. A total of 60,439 peptides were generated in the present study, among which 58,762 peptides were unique. Subsequently, the peptides were further analyzed, and the result demonstrated that a total of 6,864 proteins were identified, of which 6,822 proteins were quantified (Fig. S2). To further investigate the possible regulatory gene networks by adriamycin, DEPs (differentially expressed proteins) between HL60 and HL60/R cells were analyzed. The result showed that a total of 3,241 proteins were differentially expressed based on the criteria of a fold change > 2 and a p value < 0.05 in HL60/R compared to HL60. Among the proteins, 1,686 DEPs exhibited up-regulation, while 1,555 DEPs were down-regulated in HL60/R in comparison with HL60 (Fig. 1A). Additionally, the DEPs and their expression levels between HL60 and HL60/R cells were showed by volcano plot, scatter plot and hierarchical clustering (Fig. 1B-D). These results indicated that many proteins were significantly changed in adriamycin-resistant cell line HL60/R.

### Pathway enrichment analysis of deps

**Fig. 2:**
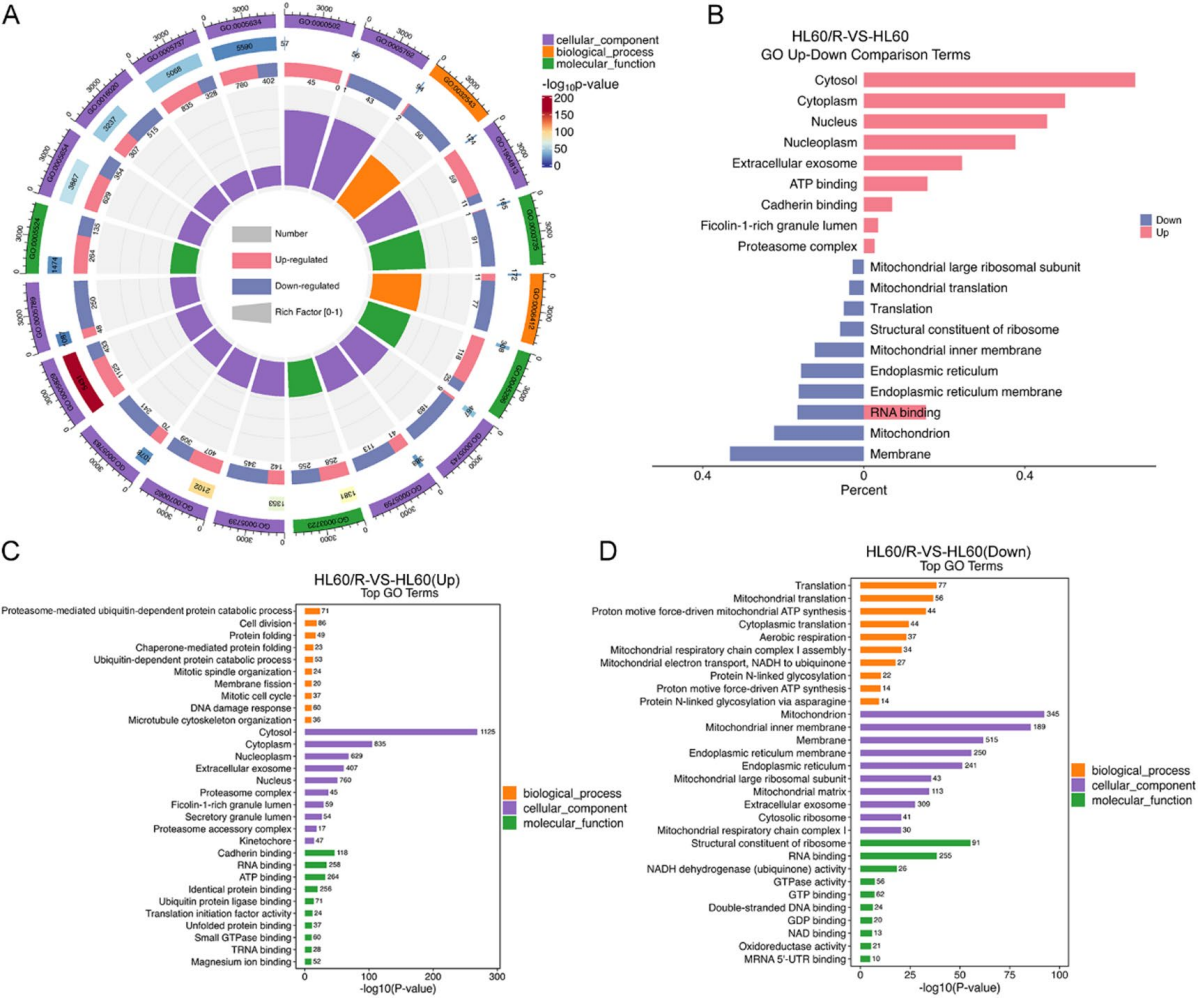
GO enrichment analysis of DEPs between HL60 and HL60/R cells. (**A**) GO enrichment analysis of the DEPs between HL60 and HL60/R cells showed the top 20 significantly enriched terms. (**B**) GO enrichment analysis of the up-regulated or down-regulated DEPs of HL60/R in comparison with HL60 cell showed the top 10 significantly enriched up-regulated or down-regulated terms. (**C**) GO enrichment analysis of up-regulated DEPs from HL60/R in comparison with HL60 cell. Y-axis showed the catalogs of biological process, cellular component and molecular function. The number in a particular term represented the actual number of DEPs. (D) GO enrichment analysis of down-regulated DEPs from HL60/R in comparison with HL60 cell.

**Fig. 3:**
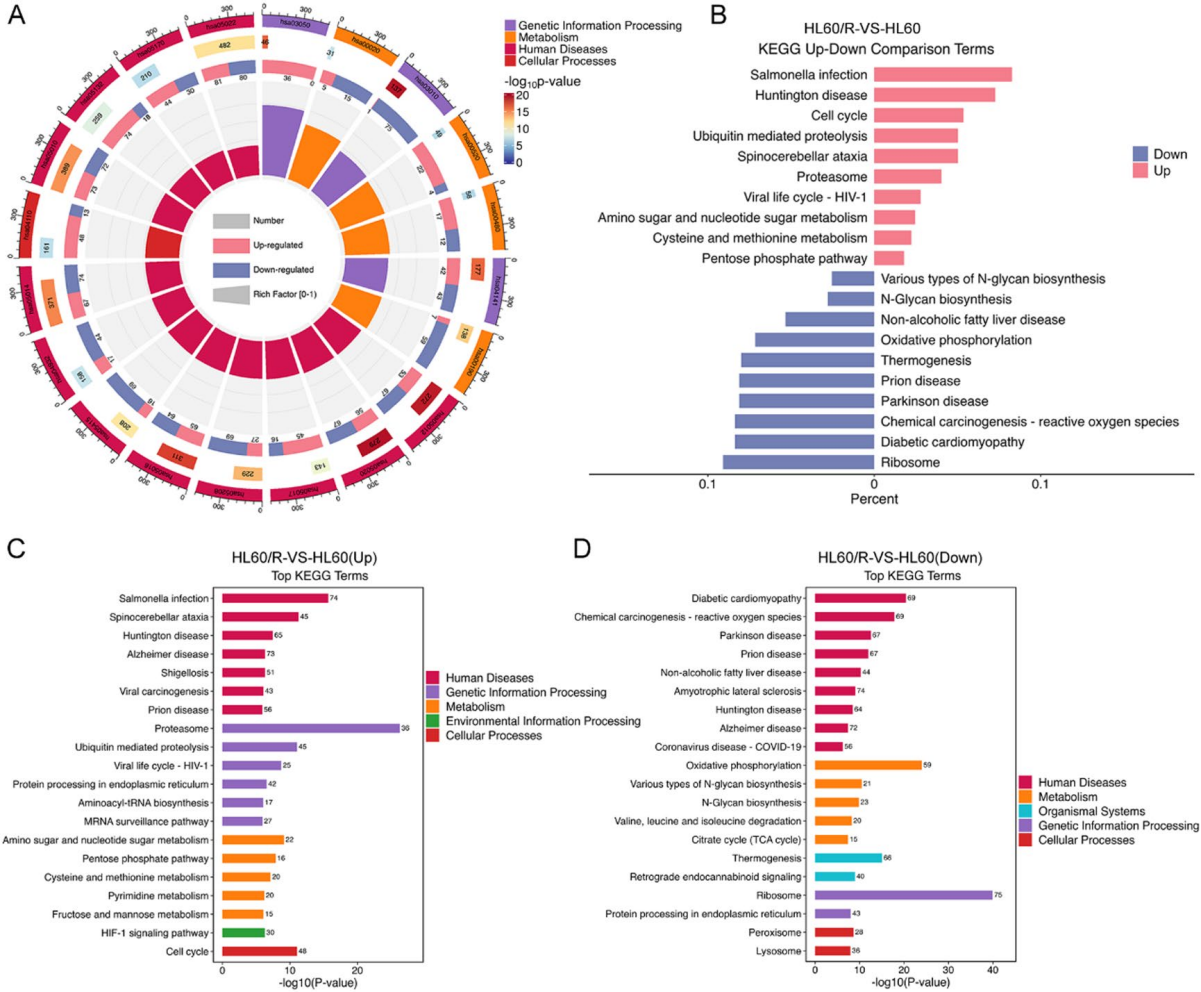
KEGG enrichment analysis of DEPs between HL60 and HL60/R cells. (**A**) KEGG enrichment analysis of the DEPs between HL60 and HL60/R cells showed the top 20 significantly enriched pathway terms. (**B**) KEGG enrichment analysis of the up-regulated or down-regulated DEPs of HL60/R in comparison with HL60 cell showed the top 10 significantly enriched up-regulated or down-regulated pathway terms. (**C**) KEGG enrichment analysis of up-regulated DEPs from HL60/R in comparison with HL60 cell. The numbers represented the actual number of DEPs that were classified in a particular pathway. (**D**) KEGG enrichment analysis of down-regulated DEPs from HL60/R in comparison with HL60 cell.

**Fig. 4:**
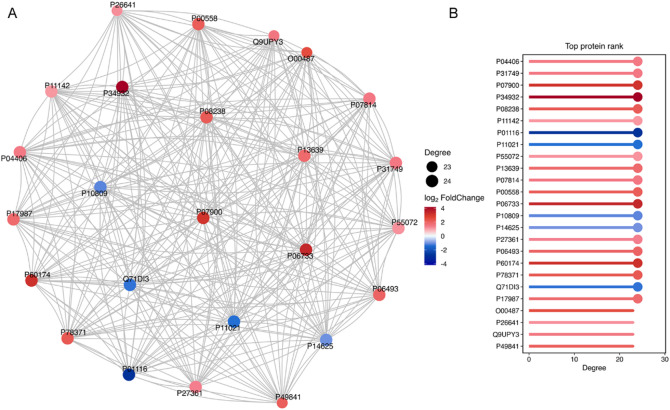
PPI network of the DEPs between HL60 and HL60/R cells. (**A**) The interaction network diagram of top 25 proteins with connectivity. The circles indicated DEPs, and red circles represented up-regulated proteins and blue circles represented down-regulated proteins. The size of the circle represented the level of connectivity, and the larger the circle is, the higher the connectivity is. (**B**) The expression histogram of top 25 proteins with connectivity.

To determine the possible functions of the DEPs between HL60 and HL60/R cells, GO classification enrichment analysis of all obtained DEPs was first performed. The results showed the top 20 significantly enriched terms, which including BPs such as translation and mitochondrial translation, CCs such as cytosol, extracellular exosome and mitochondrion, and MFs including RNA binding, cadherin binding and ATP binding (Fig. 2A). Next, we analyzed the up-regulated and down-regulated DEPs by GO classification, and the results demonstrated that the up-regulated DEPs were primary involved in cytosol, nucleus, ATP binding and RNA binding, while the down-regulated DEPs were primary association with membrane, mitochondrion, endoplasmic reticulum and translation (Fig. 2B). Furthermore, GO enrichment analysis of up-regulated and down-regulated DEPs according to the categories of BPs, CCs and MFs were performed respectively, and the top 30 significantly enriched up-regulated or down-regulated pathway terms were displayed in Fig. 2C-D. To further gain an insight into the functional and biological processes of the DEPs, KEGG pathway enrichment analysis was also carried out. The KEGG analysis of all DEPs indicated that 110 signal pathways were significantly enriched, and the top 20 significantly enriched pathways, including citrate cycle (TCA cycle), oxidative phosphorylation, cell cycle and glutathione metabolism were shown in Fig. 3A. Then the up-regulated and down-regulated DEPs were also analyzed by KEGG, and the results showed that the up-regulated DEPs were primary involved in salmonella infection, cell cycle and ubiquitin mediated proteolysis, while the down-regulated DEPs were primary association with ribosome, diabetic cardiomyopathy and oxidative phosphorylation (Fig. 3B). Additionally, KEGG analysis of up-regulated and down-regulated DEPs according to the categories were performed respectively, and the top 20 significantly enriched up-regulated or down-regulated pathway terms were shown in Fig. 3C-D. Meanwhile, to obtain the interaction network of DEPs, the top 25 DEPs with connectivity were selected for drawing a protein-protein interaction (PPI) network based on the STRING database. Among the DEPs, 20 proteins were up-regulated, and the remaining 5 proteins were down-regulated (Fig. 4A-B).

### Untargeted metabolomics data analysis

**Fig. 5:**
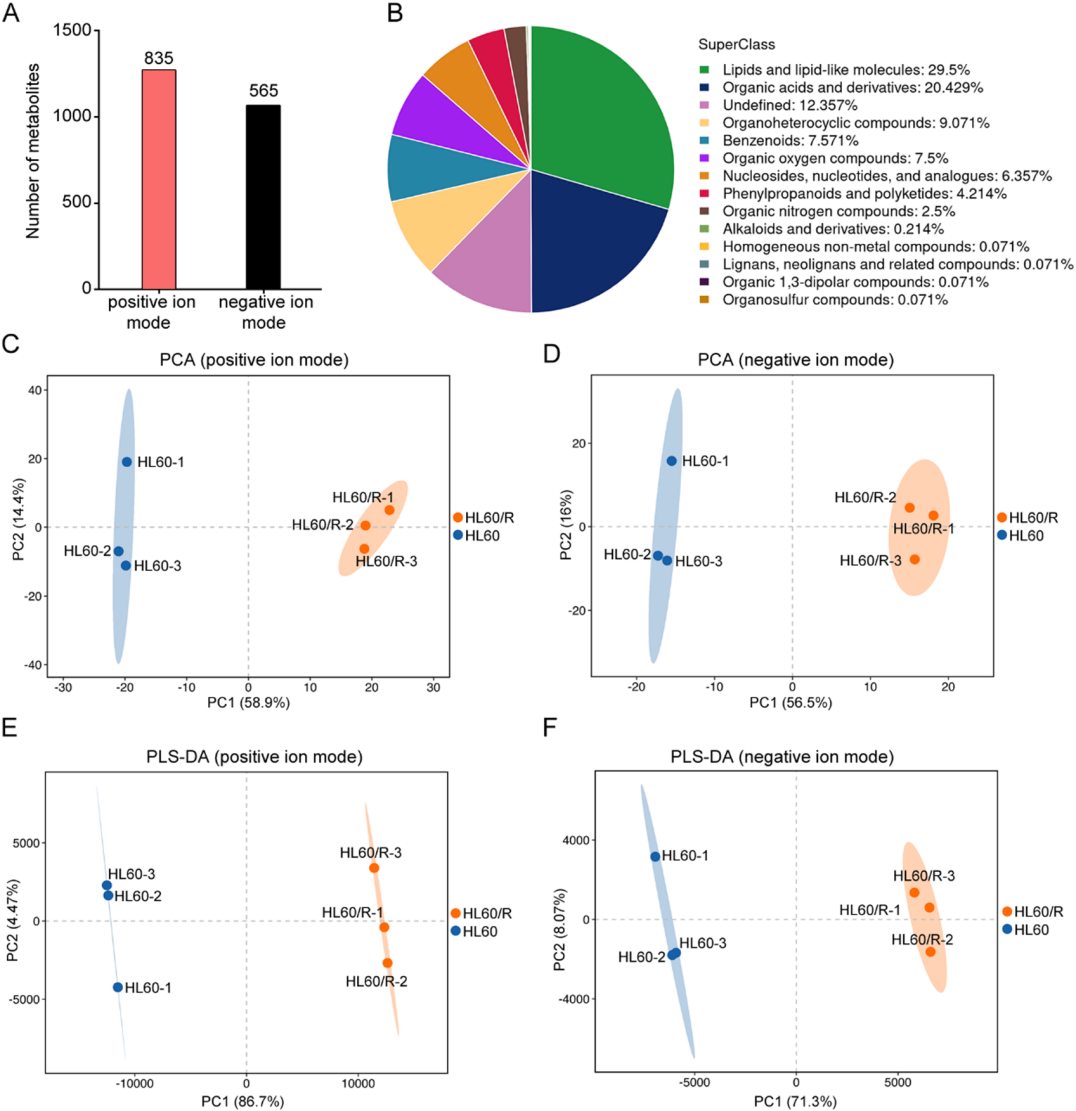
Identification of metabolites and multivariate statistical analysis between HL60 and HL60/R cells. (**A**) The numbers of identified metabolites in positive and negative ion modes. (**B**) The proportion of identified metabolites in each kind of chemical classification. (**C**) PCA score plot of the identified metabolites in positive ion mode. (**D**) PCA score plot of the identified metabolites in negative ion mode. (**E**) PLS–DA score plot of the identified metabolites in positive ion mode. (**F**) PLS–DA score plot of the identified metabolites in negative ion mode.

**Fig. 6:**
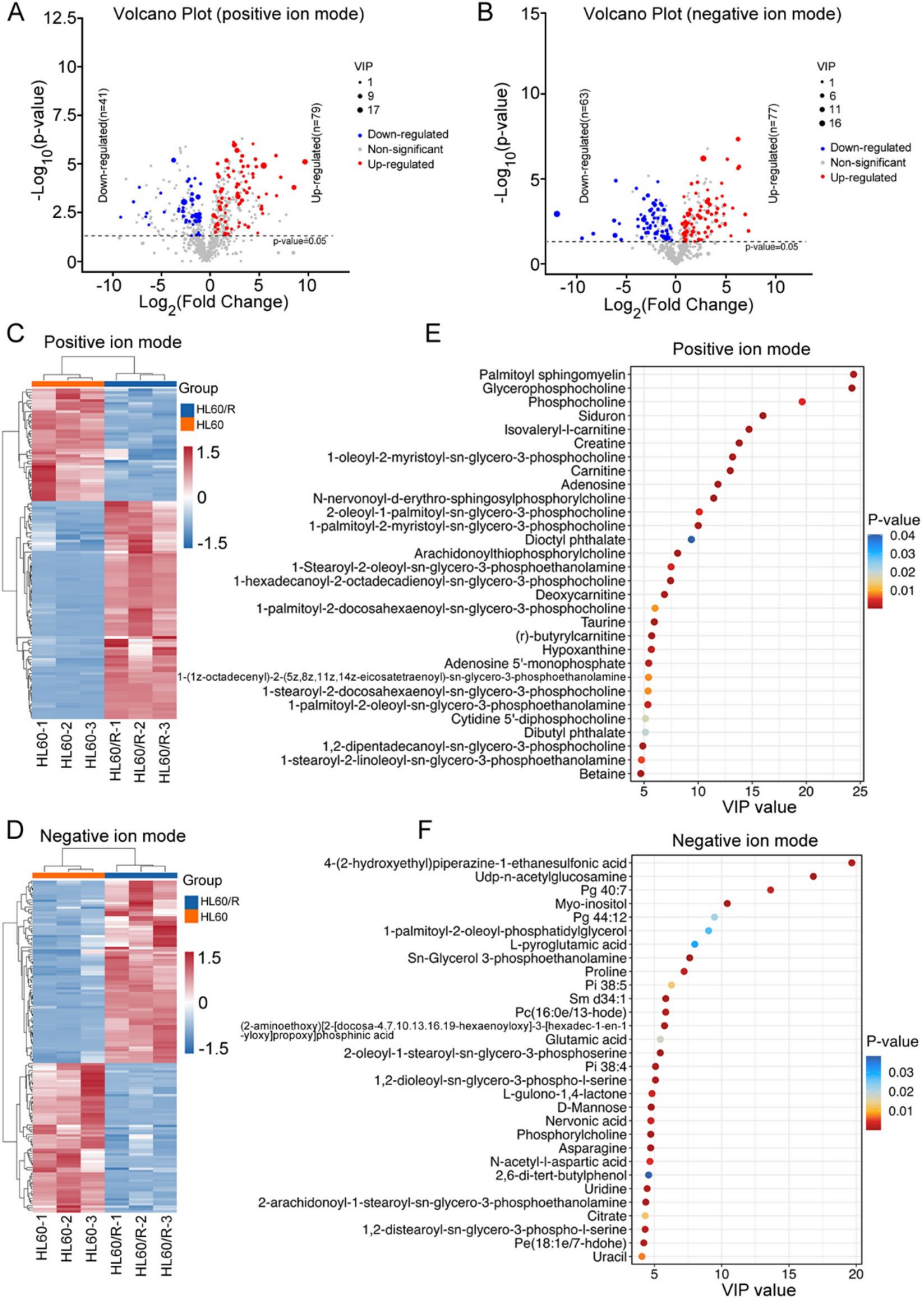
Comprehensive analysis of untargeted metabolomics between HL60 and HL60/R cells. (**A**) Volcano plot indicated the DEMs between HL60 and HL60/R cells in positive ion mode. The red and blue points represented significantly up-regulated and down-regulated metabolites, respectively, while the gray points represented equally expressed metabolites. (**B**) Volcano plot indicated the DEMs between HL60 and HL60/R cells in negative ion mode. The red and blue points represented significantly up-regulated and down-regulated metabolites, respectively, while the gray points represented equally expressed metabolites. (**C**) Hierarchical clustering indicated the DEMs and their expression levels in positive ion mode. Red represented an up-regulation, and blue represented a down-regulation. (**D**) Hierarchical clustering indicated the DEMs and their expression levels in negative ion mode. Red represented an up-regulation, and blue represented a down-regulation. (**E**) The top 30 metabolites with high discriminatory accuracy ranked by VIP value in positive ion mode. (**F**) The top 30 metabolites with high discriminatory accuracy ranked by VIP value in negative ion mode.

To identify the significantly changed metabolite profiles between HL60 and HL60/R cells, we performed untargeted metabolomics analysis using UHPLC-Q-TOF/MS. In the analysis of metabolomic data, sum normalization and Pareto (PAR) scaling were applied to enhance data comparability and reliability. Additionally, cell confluence was controlled throughout the experiment to minimize its impact on metabolic profiles. A total of 1,400 metabolites were detected, including lipids and lipid-like molecules, organic acids and derivatives, organoheterocyclic compounds, benzenoids and some others (Fig. 5A-B). Subsequently, multivariate statistical analysis between HL60 and HL60/R cells was performed. The PCA score plots of the identified metabolites illustrated that HL60/R group was different from control group in both positive and negative ion modes, indicating that the results were credible (Fig. 5C-D). Meanwhile, the results of PLS-DA model also confirmed that the HL60/R and the control group were well separated in both positive and negative ion modes, which indicated a significant difference between the two groups (Fig. 5E-F). To further screen out the potential metabolic biomarkers in adriamycin-resistant cell line HL60/R, DEMs between HL60 and HL60/R cells were analyzed. A total of 120 DEMs were detected, of which 79 metabolites exhibited up-regulation, and 41 metabolites displayed down-regulation based on the criteria of a VIP value > 1 and a p value < 0.05 in HL60/R compared to HL60 under positive ion mode (Fig. 6A). At the same time, 140 DEMs were identified, of which 77 were up-regulated and 63 were down-regulated based on the criteria of a VIP value > 1 and a p value < 0.05 in HL60/R group when compared to the control group under negative ion mode (Fig. 6B). The hierarchical clustering plot provided an overall metabolic profile of all samples, and the results of the present study showed a considerable variability in metabolomic expression between the two groups in both positive and negative ion modes (Fig. 6C-D). Furthermore, the top 30 significantly changed metabolites with high discriminatory accuracy ranked by VIP value in positive ion mode such as palmitoyl sphingomyelin, glycerophosphocholine, phosphocholine and siduron were displayed in Fig. 6E. And the top 30 significantly changed metabolites with high discriminatory accuracy ranked by VIP value in negative ion mode such as 4-(2-hydroxyethyl) piperazine-1-ethanesulfonic acid, Udp-n-acetylglucosamine, Pg 40:7 and Myo-inositol were shown in Fig. 6F. Collectively, these findings demonstrate a substantial alteration in the metabolic profile of HL60 cells upon acquisition of adriamycin resistance.

### Metabolic pathway enrichment analysis

**Fig. 7:**
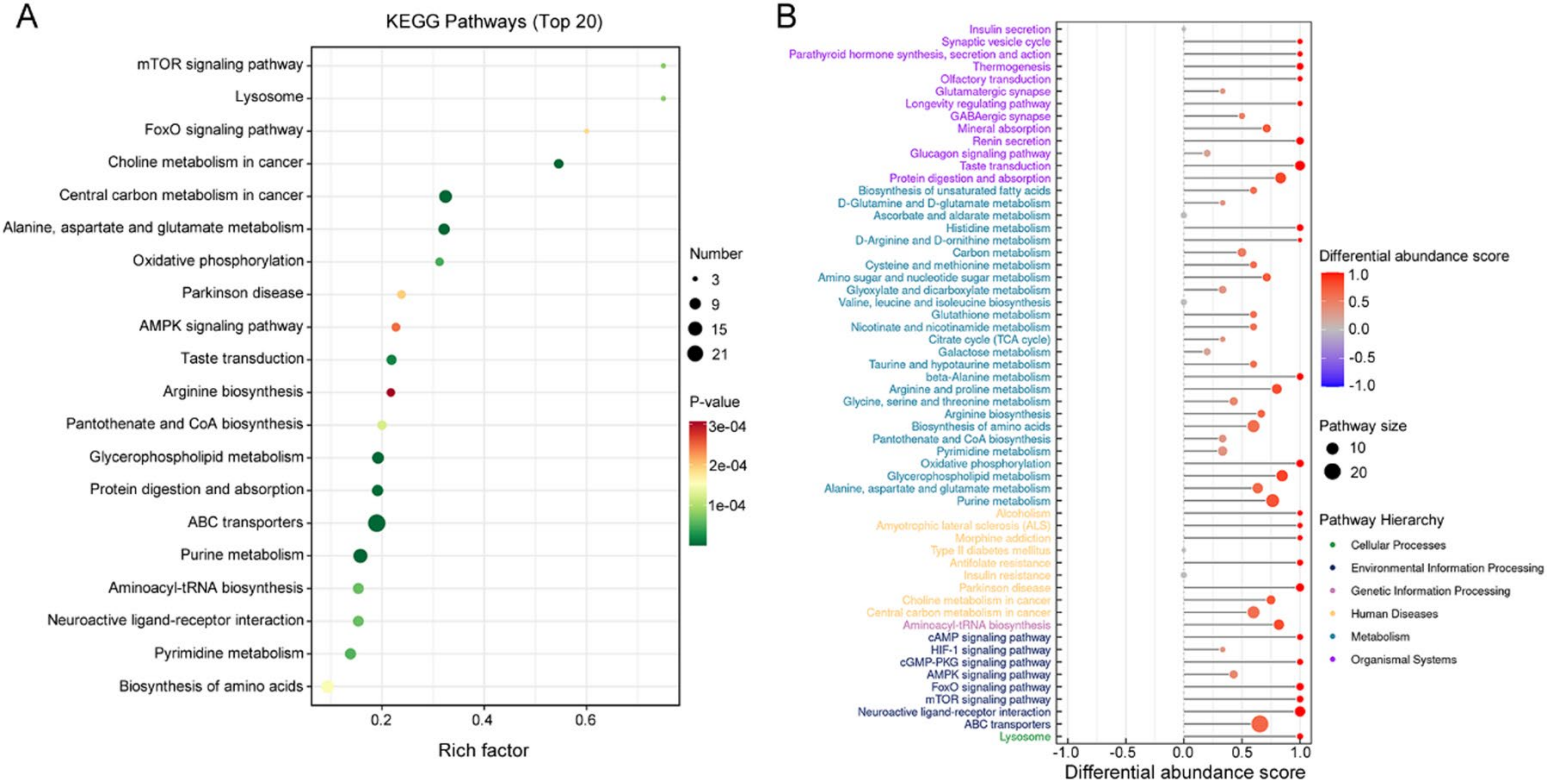
Pathway enrichment analysis of DEMs between HL60 and HL60/R cells. (**A**) KEGG enrichment analysis of the DEMs between HL60 and HL60/R cells showed the top 20 significantly enriched metabolic pathway terms. (**B**) Pathway analysis of metabolomic alterations based on the differential abundance scores of metabolites between HL60 and HL60/R cells. A score of 1 denoted that all metabolites in a particular pathway are increased in HL60/R cells compared to HL60 cells, while a score of -1 denoted that all metabolites in a particular pathway are decreased.

To gain a deeper understanding of the biological processes of DEMs, metabolic pathway enrichment analysis was conducted based on the KEGG database. The pathway analysis revealed that a total of 58 signal pathways were significantly enriched, and the top 20 significantly enriched pathways were displayed in Fig. 7A. Among these pathways, we found that alanine, aspartate and glutamate metabolism, arginine biosynthesis, pantothenate and CoA biosynthesis, glycerophospholipid metabolism, purine metabolism, aminoacyl-tRNA biosynthesis, pyrimidine metabolism, and biosynthesis of amino acids were markedly perturbed in HL60/R cells, suggesting these enriched pathways may involve in the pathogenesis of adriamycin resistance (Fig. 7A). In addition, differential abundance score analysis was used to further analyze metabolomics data of the two groups. The results showed that multiple metabolic pathways, particularly histidine metabolism, beta-alanine metabolism, arginine and proline metabolism, biosynthesis of amino acids, purine metabolism, alanine, aspartate and glutamate metabolism, and glycerophospholipid metabolism were significantly altered pathways (Fig. 7B). Overall, metabolomics analysis elucidated that adriamycin-resistant cell lines HL60/R were metabolically dysregulated.

### Integration analysis of proteomics and metabolomics

**Fig. 8:**
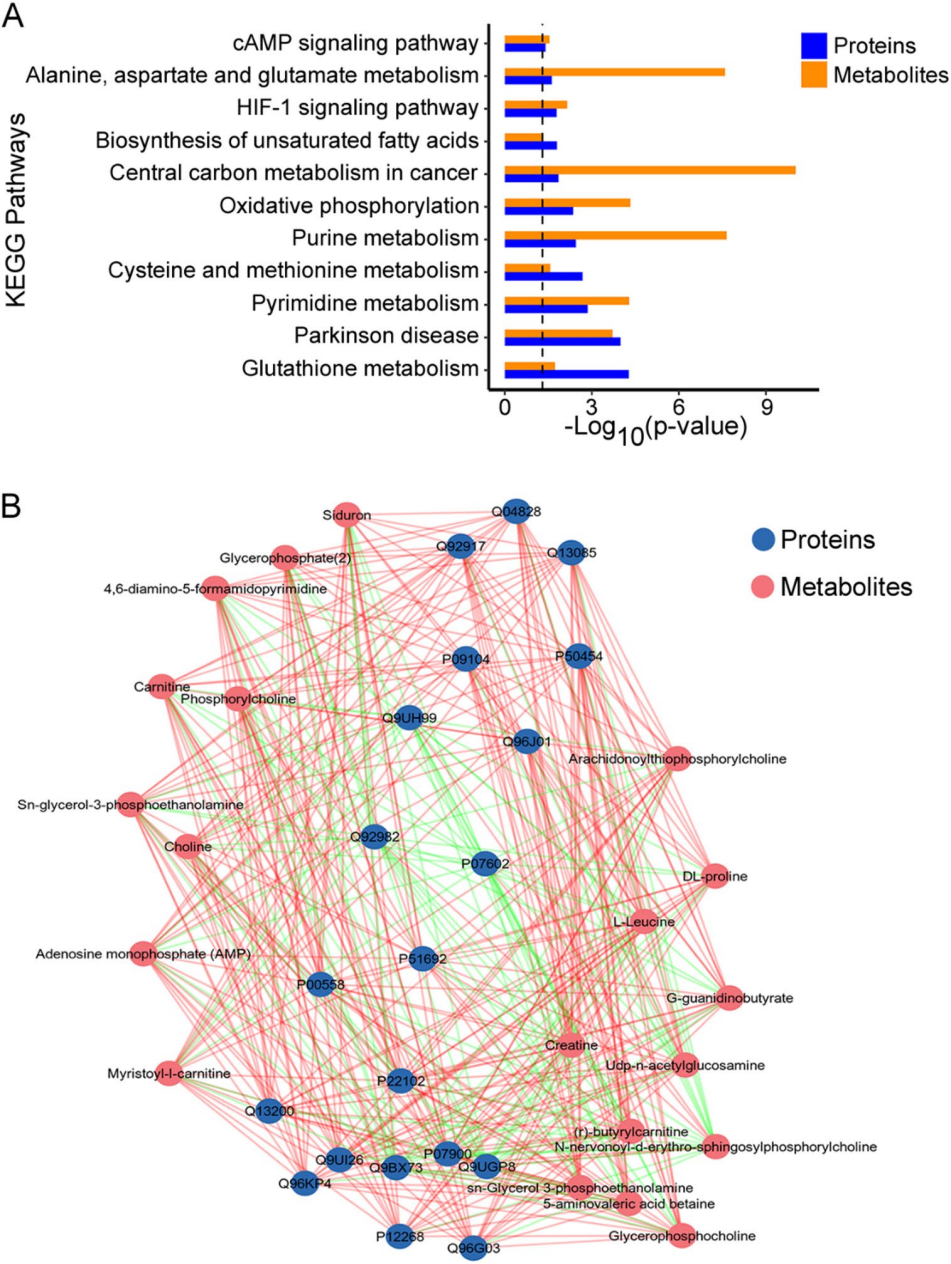
Integrated analysis of proteomics and metabolomics between HL60 and HL60/R cells. (**A**) KEGG co-enrichment analysis of DEPs and DEMs showed significantly altered pathways between HL60 and HL60/R cells. (**B**) A correlation network diagram of the top 20 DEPs and the top 20 DEMs. The blue circles represented proteins, and the red circles represented metabolites. The red line represented a positive correlation, and the green line represented a negative correlation. The thickness of the line represented the level of the correlation coefficient.

To obtain a comprehensive profile and identify the relationships between proteins and metabolites of the adriamycin-resistant and control groups, we performed an integrated analysis of proteomic and metabolomic data. Functional enrichment analysis showed that the DEPs and DEMs were mostly enriched in cAMP (cyclic-adenosine monophosphate) signaling pathway, HIF-1 (hypoxia-inducible factor 1) signaling pathway and oxidative phosphorylation. Meanwhile, metabolism pathways were mainly involved in amino acid metabolism, such as alanine, aspartate and glutamate metabolism, cysteine and methionine metabolism and glutathione metabolism. In addition, purine metabolism, pyrimidine metabolism, central carbon metabolism in cancer and biosynthesis of unsaturated fatty acids were also significantly enriched (Fig. 8A). Furthermore, the top 20 DEPs and DEMs were selected out according to the p-values, and the correlation between proteins and metabolites was calculated by Pearson correlation analysis. Subsequently, a correlation network diagram of the top 20 DEPs and DEMs was drawn (Fig. 8B). These results suggested that a possible regulatory crosstalk of amino acid metabolism, which might provide a potential approach to inhibit adriamycin resistance in HL60 cells.

### Western blot analysis

**Fig. 9:**
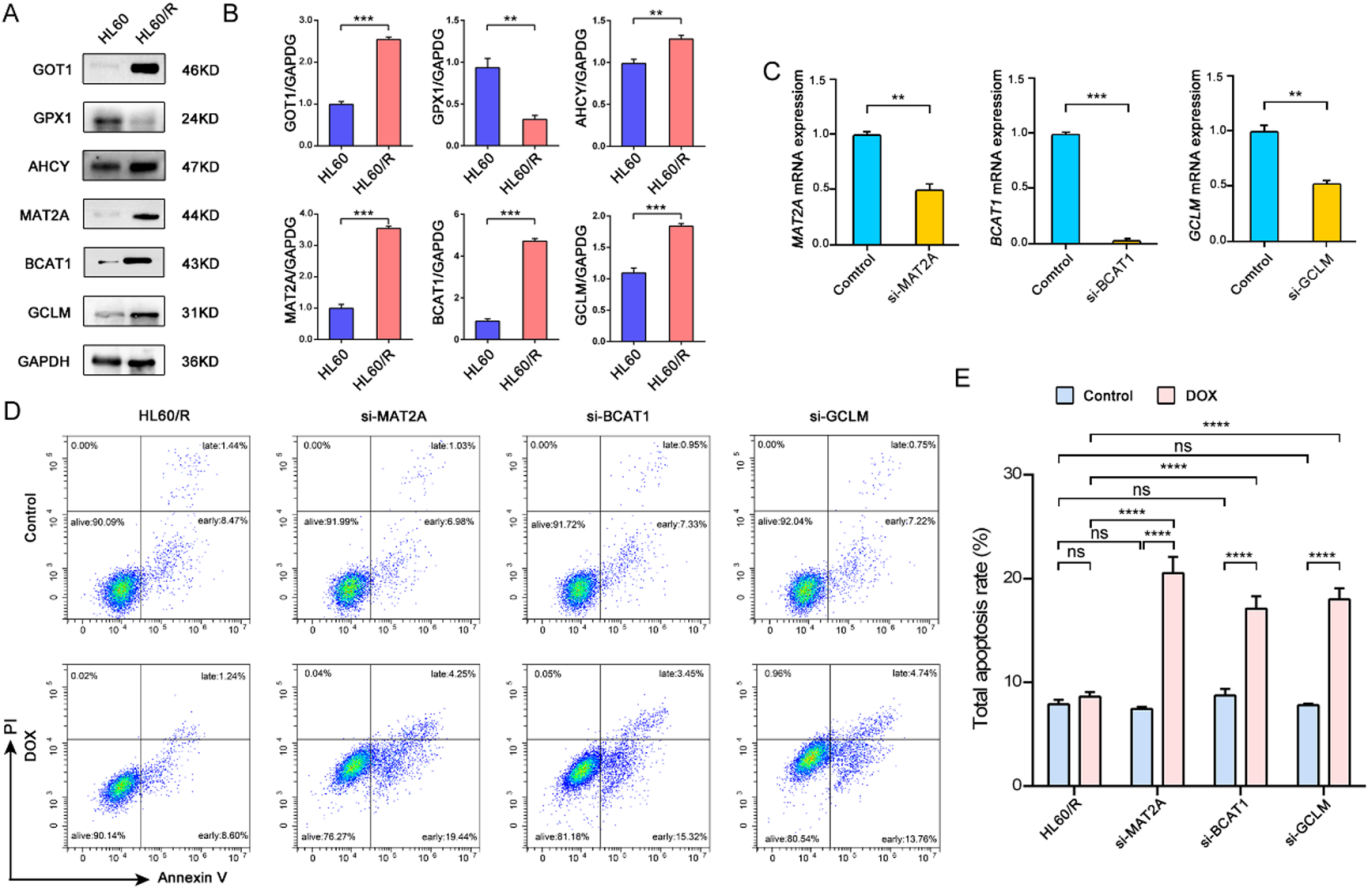
Effects of amino acid metabolism in HL60/R cells. (**A**) Western blot analysis of 6 selected DEPs between HL60 and HL60/R cells. GAPDH was used as internal reference. (**B**) Proteins expression level compared with GAPDH. (**C**) qRT-PCR of si-MAT2A, si-BCAT1 and si-GCLM HL60/R cells. (**D**) Apotosis assay of si-MAT2A, si-BCAT1 and si-GCLM HL60/R cells by Flow cytometry. (**E**) Apotosis analysis of si-MAT2A, si-BCAT1 and si-GCLM HL60/R cells from the Flow cytometry data.**p < 0.01, ***p < 0.001, ****p < 0.0001.

To confirm the accuracy of the proteomic analysis results of this study, we selected six interested proteins related to amino acid metabolism, which including GOT1 (glutamic-oxaloacetic transaminase 1), AHCY (adenosylhomocysteinase), MAT2A (methionine adenosyltransferase 2 A), BCAT1 (branched chain amino acid transaminase 1), GCLM (glutamate-cysteine ligase modifier subunit) and GPX1 (glutathione peroxidase 1), for western blot analysis. The results of the western blot showed that the expression levels of GOT1, AHCY, MAT2A, BCAT1 and GCLM were upregulated, while the expression level of GPX1 was downregulated in HL60/R in comparison with HL60 (Fig. 9A-B). The western blot results showed a high degree of consistency with the proteomic data, which indicated amino acid metabolism was disturbed in adriamycin-resistant HL60 cells.

To further verify the role of amino acid metabolism in HL60 cell drug resistance, MAT2A, BCAT1 and GCLM were selected for small interfering RNA experiments for subsequent experimental analysis (Fig. 9C). Flow cytometry was used to detect the apoptosis levels after si-MAT2A, si-BCAT1 or si-GCLM transfecting in HL60/R cells for 48 h (Fig. 9D, E). The results exhibited that apoptosis rates significantly promoted in HL60/R when MAT2A, BCAT1 or GCLM were interfered (Fig. 9C-E).

## Discussion

As a common acute leukemia in adults, AML is a lethal hematopoietic neoplasm in the absence of treatment. The cause is complications of pancytopenia, namely infection and bleeding. The traditional treatment for AML is induction chemotherapy and post-remission (consolidation) therapy. Induction therapy aims to achieve a complete remission, followed by consolidation therapy with the goal of eliminating residual lesions with a view to achieving a durable cure and remission^26^. Nonetheless, after receiving conventional chemotherapy, about one-third of patients with AML do not achieve complete remission and subsequently develop drug-resistant AML, which exhibits aggressive behavior against all normal treatment strategies^27^. Adriamycin is a widely used chemotherapeutic agent in clinical practice, but its efficacy is usually limited. In addition to its toxicity to normal cell growth, drug resistance is the major obstacle for its treatment of hematological malignancies^28^. Hence, to address this challenge, the regulatory mechanisms underlying adriamycin resistance in AML need to be further investigated. In the current study, the combined analysis of proteomics and metabolomics was used to investigate the regulatory mechanism of adriamycin resistance in acute myeloid leukemia cells. We unveiled that amino acid metabolism was significantly changed in adriamycin resistance cells. The findings of this study enriched our understanding of adriamycin resistance and might have potential implications for diagnosis and treatment in acute myeloid leukemia.

The combined KEGG pathway enrichment analysis suggested that cAMP signaling pathway, HIF-1 signaling pathway and oxidative phosphorylation may be actively implicated in the adriamycin resistance of acute myeloid leukemia cells. In fact, in Estrogen receptor (ER)-positive breast cancer, reactivation of cAMP pathway can overcome tamoxifen resistance^29^. Mechanistic investigations unveil that the addition of cAMP analogs or knockdown of PDE4D (phosphodiesterase4D) can gradually induce the production of cAMP, which in turn activates endoplasmic reticulum stress, increases the phosphorylation of JNK (c-Jun N-terminal kinase) and p38 MAPK (mitogen-activated protein kinases) and apoptosis, thereby overcoming tamoxifen resistance^29^. Meanwhile, previous study has shown that cAMP signaling pathway was associated with drug resistance in lung cancer^30^, and this pathway can be regulated by ADCY1 (Adenylate cyclase 1), which catalyzes ATP to cyclic AMP^30^. Another study clarified that the cAMP signaling pathway was involved in methotrexate resistance in colorectal cancer through the KCNQ1OT1/miR-760/PPP1R1B (protein phosphatase 1 regulatory subunit 1B) axis^31^. It is well known that the HIF-1 signaling was activated in most human tumors due to hypoxic environment and epigenetic mechanisms, which can lead to physiological changes that ultimately cause therapeutic resistance^32^. For example, in gastric cancer, HIF-1α can induce anti-cisplatin effects through dysregulating microRNAs and lncRNAs, upregulating inflammatory factors, and inhibiting the activities of p53 and NF-kappaB^33^. In lung adenocarcinoma, Polyphyllin I can effectively counteract gefitinib resistance by facilitating HIF-1α /VHL (Von Hippel-Lindau) complex formation and then leading to HIF-1α degradation, ultimately inhibiting the VEGF (vascular endothelial growth factor)/VEGFR2 (vascular endothelial growth factor receptor 2)/p38 pathway and angiogenesis^34^. At the same time, research has found that oxidative phosphorylation can maintain cancer resistance and metastasis^35^. Previous literature has indicated that targeting oxidative phosphorylation using its inhibitor IM156 or metformin can inhibit the growth of ibrutinib-resistant lymphoma cells both in vitro and in mouse models^36^. Similarly, in estrogen receptor positive breast cancers, the use of oxidative phosphorylation inhibitor IACS-010759 strongly inhibited tumor growth in multiple endocrine and palbociclib resistant patient-derived xenografts^37^. Hence, we speculated that the disorder of the three pathways was directly related to the occurrence of adriamycin resistance in acute myeloid leukemia cells. Nonetheless, the precise regulatory relationship still needs to be further investigated.

Metabolic reprogramming, which derived from drug-specific therapy, assumes a crucial role in driving drug resistance in tumor cells^38^. Accumulating evidence has shown that amino acid metabolism is a crucial determinant for drug resistance, as it can support cancer cell needs, such as redox homeostasis, biomass production, epigenetic modification and energy generation^39^. In this study, certain amino acid metabolic pathways, including alanine, aspartate and glutamate metabolism, cysteine and methionine metabolism and glutathione metabolism were observed to be the most widespread and marked alteration in adriamycin resistance acute myeloid leukemia cells for the first time. Recent study has demonstrated that in colorectal cancer, metabolic disorders of alanine, aspartate and glutamate may be involved in the reduction of the sensitivity of colorectal cancer to 5-fluorouracil (5-FU) chemotherapy by hypoxia^40^. More interestingly, in pancreatic ductal adenocarcinoma, significant change in alanine, aspartate and glutamate metabolic pathway was observed after postoperative chemotherapy with nab-paclitaxel plus gemcitabine^41^. In addition, glutathione acts as an antioxidant, and if its expression levels are elevated in breast, colon, laryngeal, and lung cancer cells, it will promote their resistance to chemotherapy drug treatment^42^. Specifically, another study reported that cysteine and glutathione could be accurately monitored by a NIR (Near Infrared Ray) fluorescent probe, which can clearly assess the redox regulatory capacity of tumors and the progression of drug resistance in tumor-bearing mice^43^. Recent another study has demonstrated that in bladder cancer, cisplatin-resistant tumor cells are methionine dependent for survival and stem cell renewal^44^. Hence, to further understand the changes of amino acid metabolism in HL60/R cells, six significantly changed amino acid metabolism-related proteins GOT1, GPX1, AHCY, MAT2A, BCAT1, and GCLM were further validated through western blot. Taken together, our data support previous reports of amino acid metabolic pathways are involved in drug resistance, which suggest that intervening in amino acid metabolism may provide a meaningful pathway for diagnosis and treatment of drug resistance in acute myeloid leukemia cells.

Moreover, our study has uncovered that purine metabolism, pyrimidine metabolism, central carbon metabolism in cancer and biosynthesis of unsaturated fatty acids were also significantly changed in adriamycin resistance cells. In glioblastoma, inhibition of purine synthesis by AICAR (5-aminoimidazole-4-carboxamide ribonucleoside) can sensitize temozolomid-resistant glioblastoma cells to temozolomid-resistant chemotherapy, which means purine metabolism is involved in drug resistance^45^. In pancreatic ductal adenocarcinoma, there is a large infiltration of tumor-associated macrophages, which release pyrimidines that inhibit gemcitabine through molecular competition, leading to drug resistance^46^. Notably, the poor efficacy of antibiotic therapy, as well as the frequent emergence of antibiotic-resistant Mycobacterium tuberculosis, is closely associated with central carbon metabolism remodeling^47^. Additionally, in nude mice bearing HepG2/adriamycin xenografts and HepG2/adriamycin cells, adriamycin-loaded omega-3 unsaturated fatty acids nanoparticles can reverse hepatocellular carcinoma multidrug resistance, which indicated that unsaturated fatty acids could inhibit the development of drug resistance^48^. Thus, these data support that the above metabolic pathways were important for drug resistance. Undoubtedly, this study provides valuable resources for further study on the mechanism of adriamycin resistance in acute myeloid leukemia cells.

## Conclusion

Overall, the present study employed proteomic and metabolomic techniques to quantify and analyze the protein and metabolic profiles of HL60 and adriamycin-resistant cell line HL60/R. The proteomics analysis showed that DEPs were mainly involved in cAMP signaling pathway, HIF-1 signaling pathway and oxidative phosphorylation. An integrative analysis of proteomic and metabolomic data revealed significant disruptions in amino acid metabolism in HL60/R cells, including the pathways involved in alanine, aspartate and glutamate metabolism, cysteine and methionine metabolism, and glutathione metabolism. This study expands our understanding of the mechanisms underlying adriamycin resistance in acute myeloid leukemia cells.

## Supplementary Information

Below is the link to the electronic supplementary material.

Supplementary Material 1

Supplementary Material 2

Supplementary Material 3

Supplementary Material 4

Supplementary Material 5

Supplementary Material 6

Supplementary Material 7

Supplementary Material 8

Supplementary Material 9

Supplementary Material 10

Supplementary Material 11

Supplementary Material 12

Supplementary Material 13

Supplementary Material 14

Supplementary Material 15

## Data Availability

The LC-MS/MS mass spectrometry proteomics data have been deposited to the iProX database (http://www.iprox.org) with the iProX accession: IPX0013022001. Untargeted metabolomics data is available at the NlH Common Fund’s National Metabolomics Data Repository (NMDR) website, the Metabolomics Workbench (https://www.metabolomicsworkbench.org), the DataTrack ID was 6281.
