# Supplementary material for: Integrative analysis of proteomics and metabolomics reveals amino acid metabolism disorder in adriamycin-resistant acute myeloid leukemia cells
Source: Sci Rep. 2026 Jan 9;16:4902. doi: 10.1038/s41598-026-35675-3 (PMC12873124; doi:10.1038/s41598-026-35675-3)
Supplement: Supplementary file 4 — Supplementary Material 4 [file 41598_2026_35675_MOESM4_ESM.docx]

**Integrative analysis of proteomics** **and metabolomics reveals amino acid metabolism disorder in adriamycin-resistant** **acute myeloid leukemia cells**

Cong Li1^†^, Xue Liang^1†^, Siqi Gong^1†^, Mengmeng Fan^1^, Tianfang Shan^1^, Qiang Hong^2*^, Yanghua Tian^3*^, Zhimin Zhai^1*^

^1^ Department of Hematology, The Second Affiliated Hospital of Anhui Medical University, Hefei, 230601, China

^2^ School of Basic Medical Sciences, Anhui Medical University, Hefei, 230032, China

^3^ Department of Neurology, The Second Affiliated Hospital of Anhui Medical University, Hefei, 230601, China

*Correspondence:
Zhimin Zhai, Yanghua Tian and Qiang Hong
zzzm889@163.com, ayfytyh@126.com, hongqiang@ahmu.edu.cn

^†^These authors contributed equally to this work and share first authorship.


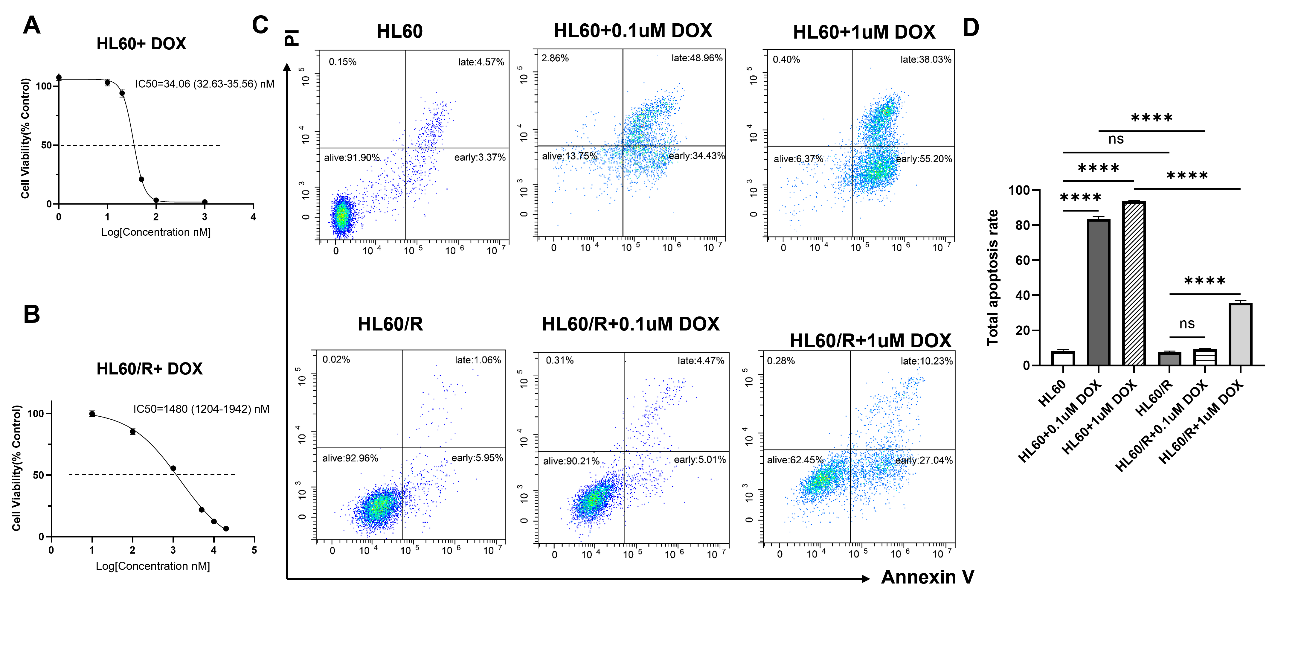


**Figure S1.** Validation of HL60/R adriamycin resistance. (A) IC50 of DOX in HL60 cell line. (B) IC50 of DOX in HL60/R cell line. (C) Assessment of apoptosis by Annexin V-APC/PI and flow cytometry in HL60 and HL60/R cells. 0.1 μM or 1 μM DOX were used to treat the cells. (D) Percentage of apoptotic cells according to the apoptosis assessment in (C). ns, no significance, ****p< 0.0001.

**
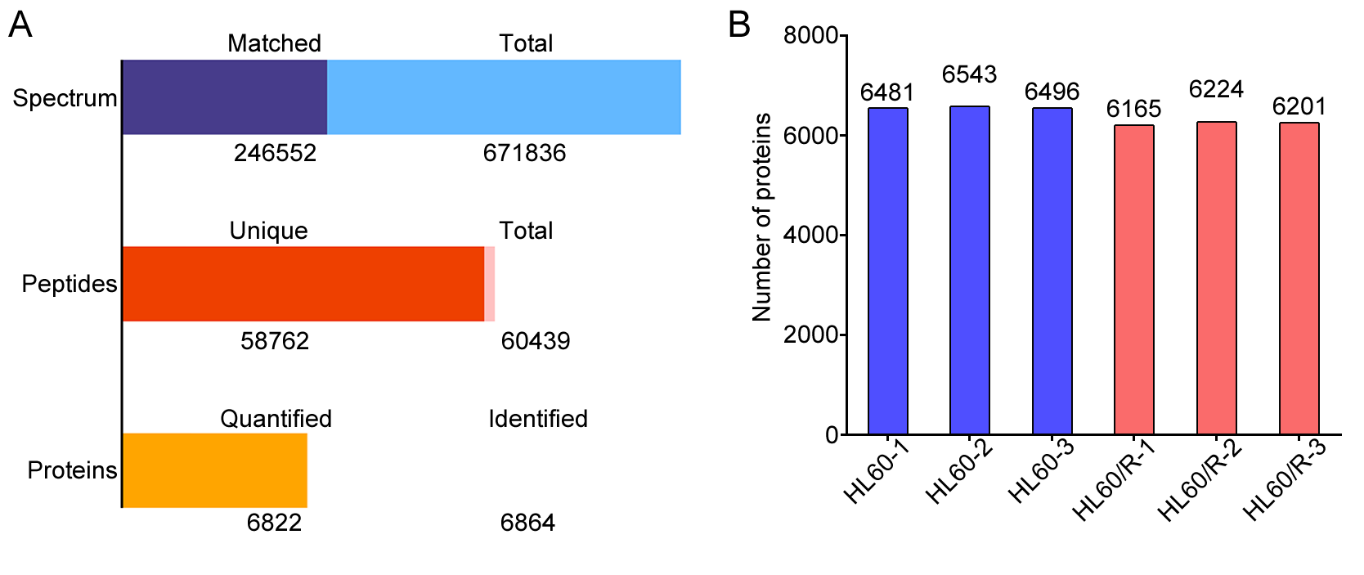
**

**Figure S2.** The proteomics data of HL60 and HL60/R cells. (A) The numbers of total and matched spectrum, total and unique peptides and identified and quantified proteins. (B) The numbers of identified proteins in HL60 and HL60/R cells.
