# Supplementary figures and images for: Integrative analysis of proteomics and metabolomics reveals amino acid metabolism disorder in adriamycin-resistant acute myeloid leukemia cells
Source: Sci Rep. 2026 Jan 9;16:4902. doi: 10.1038/s41598-026-35675-3 (PMC12873124; doi:10.1038/s41598-026-35675-3)

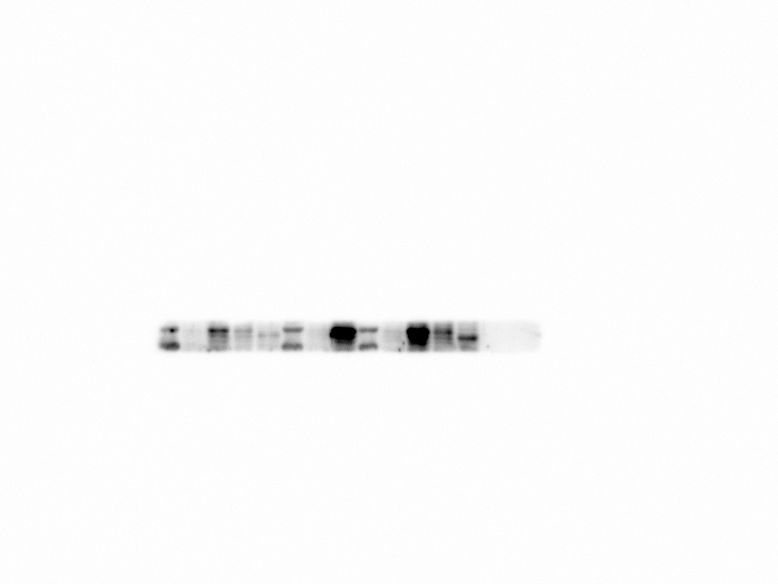

Supplement: Supplementary file 5 — Supplementary Material 5 [file 41598_2026_35675_MOESM5_ESM.png]

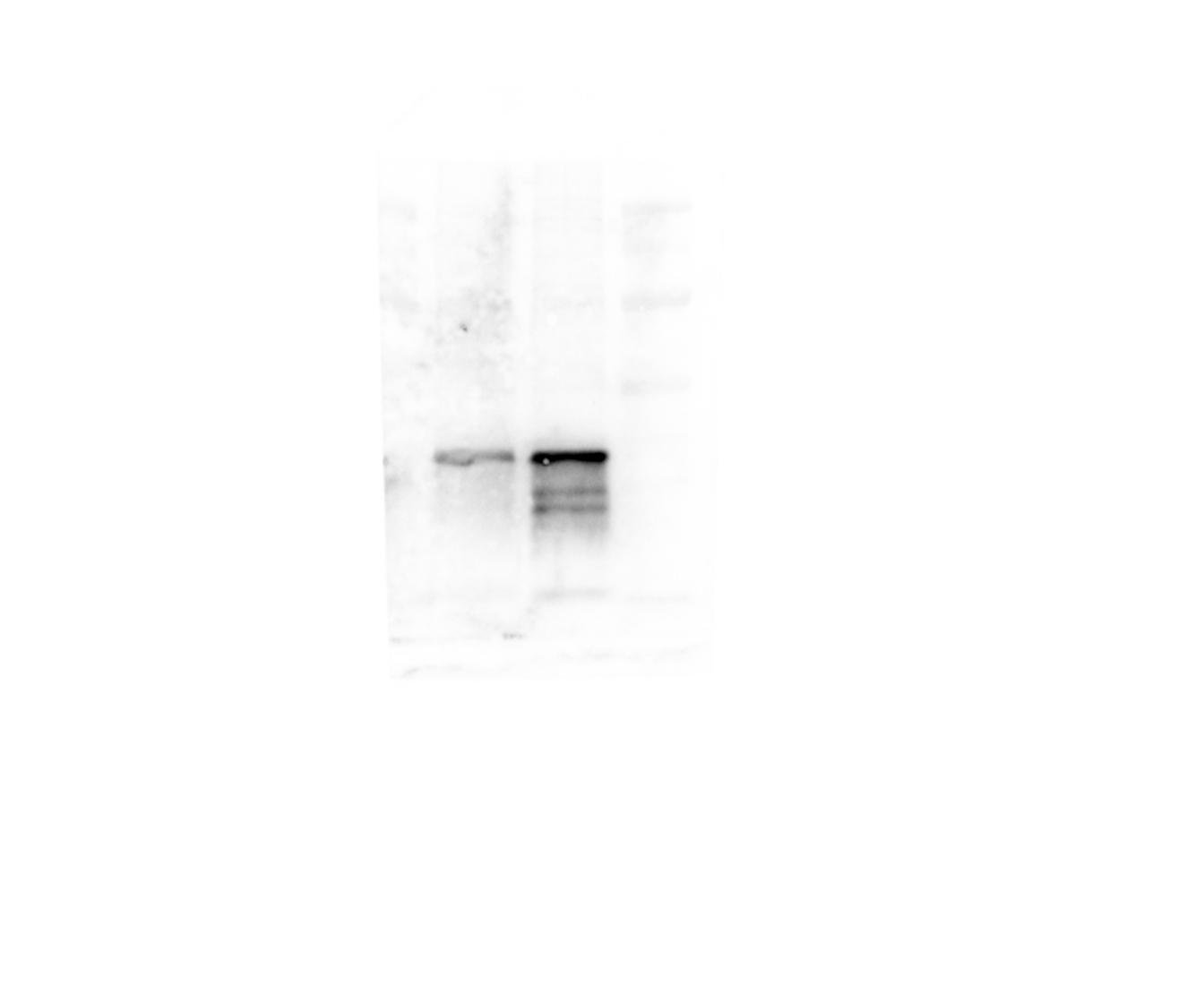

Supplement: Supplementary file 6 — Supplementary Material 6 [file 41598_2026_35675_MOESM6_ESM.tif]

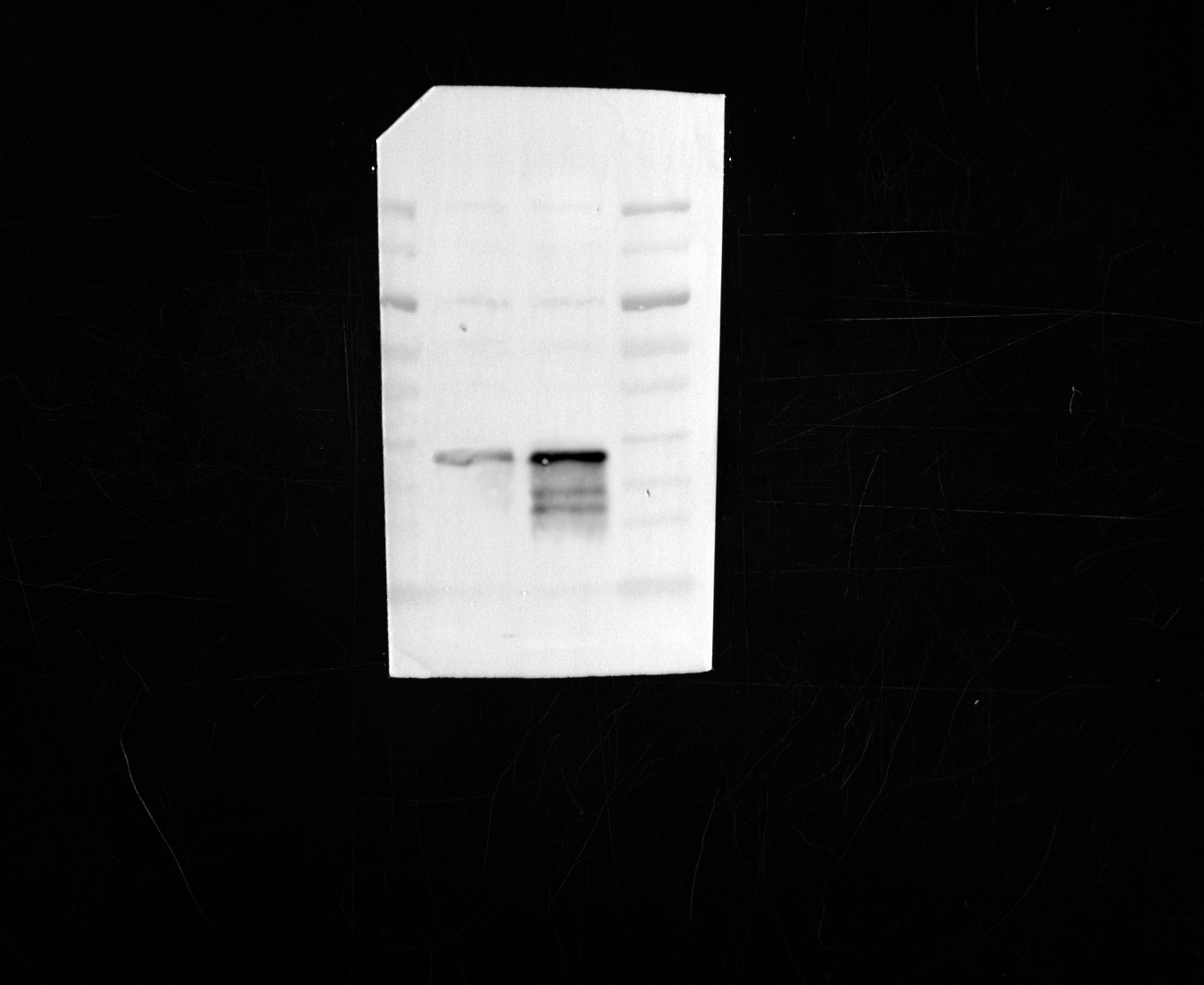

Supplement: Supplementary file 7 — Supplementary Material 7 [file 41598_2026_35675_MOESM7_ESM.tif]

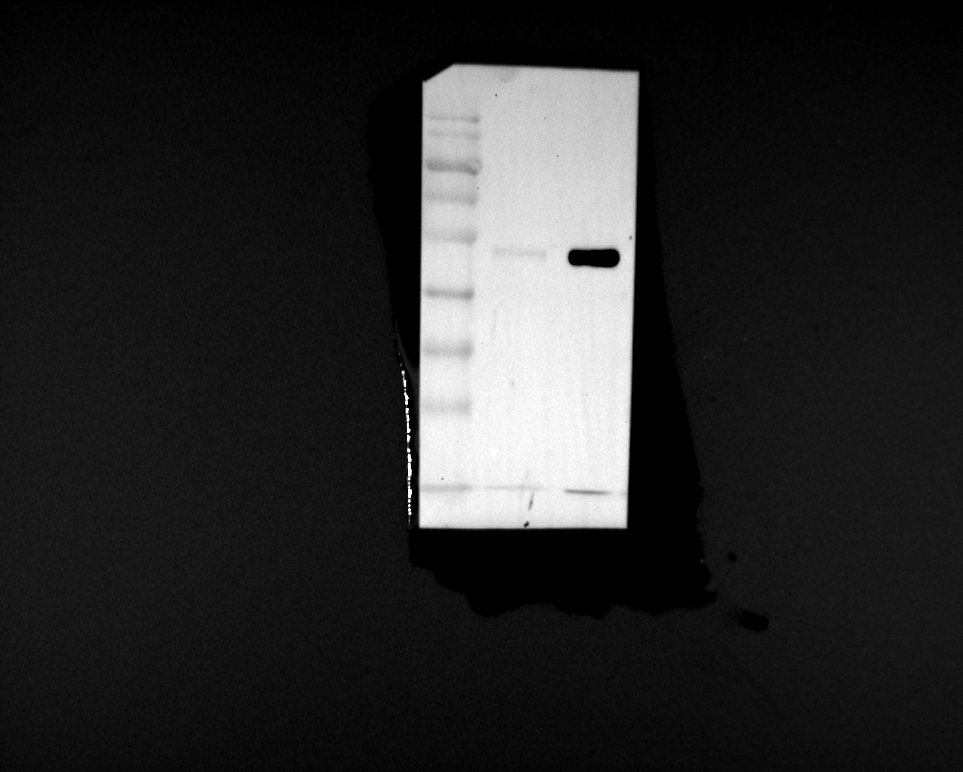

Supplement: Supplementary file 8 — Supplementary Material 8 [file 41598_2026_35675_MOESM8_ESM.tif]

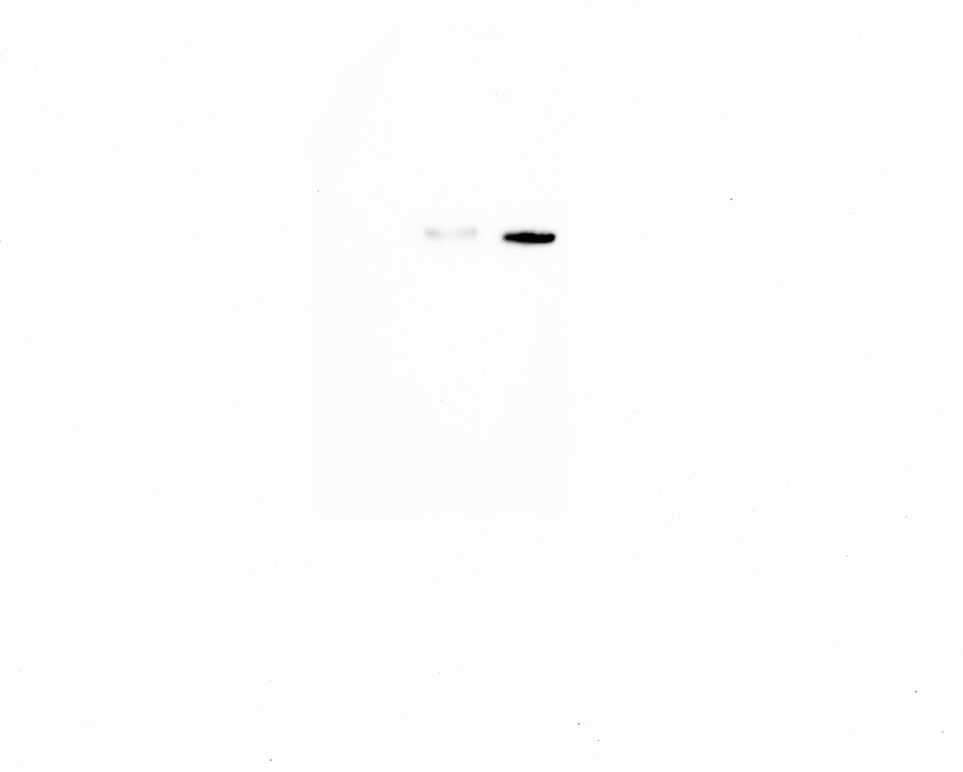

Supplement: Supplementary file 9 — Supplementary Material 9 [file 41598_2026_35675_MOESM9_ESM.tif]

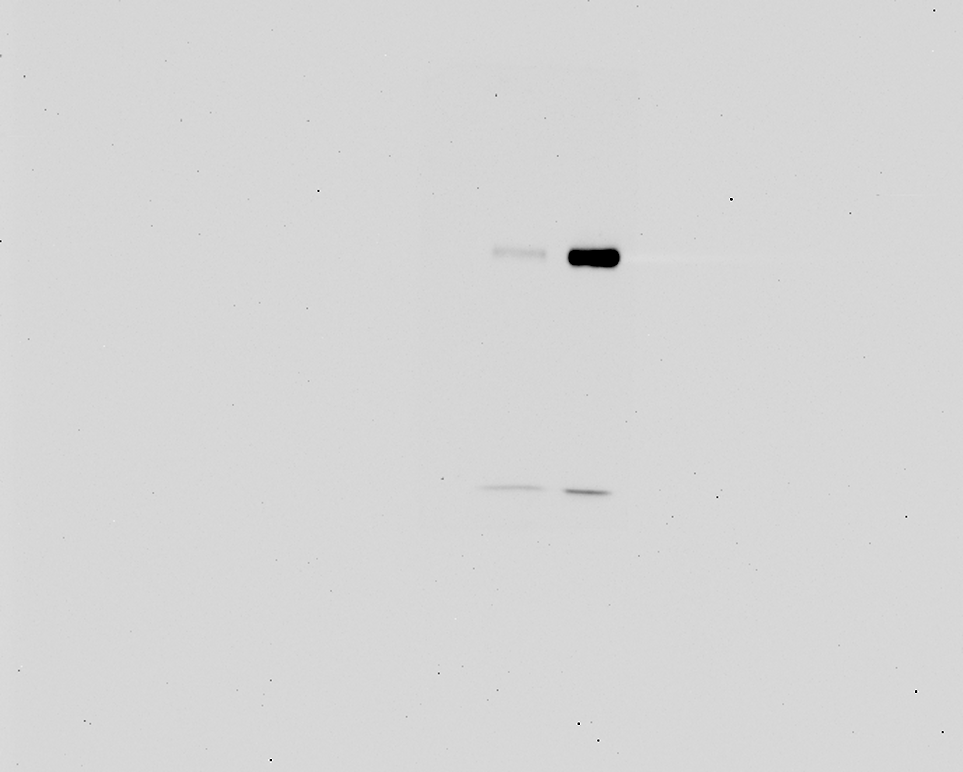

Supplement: Supplementary file 10 — Supplementary Material 10 [file 41598_2026_35675_MOESM10_ESM.tif]

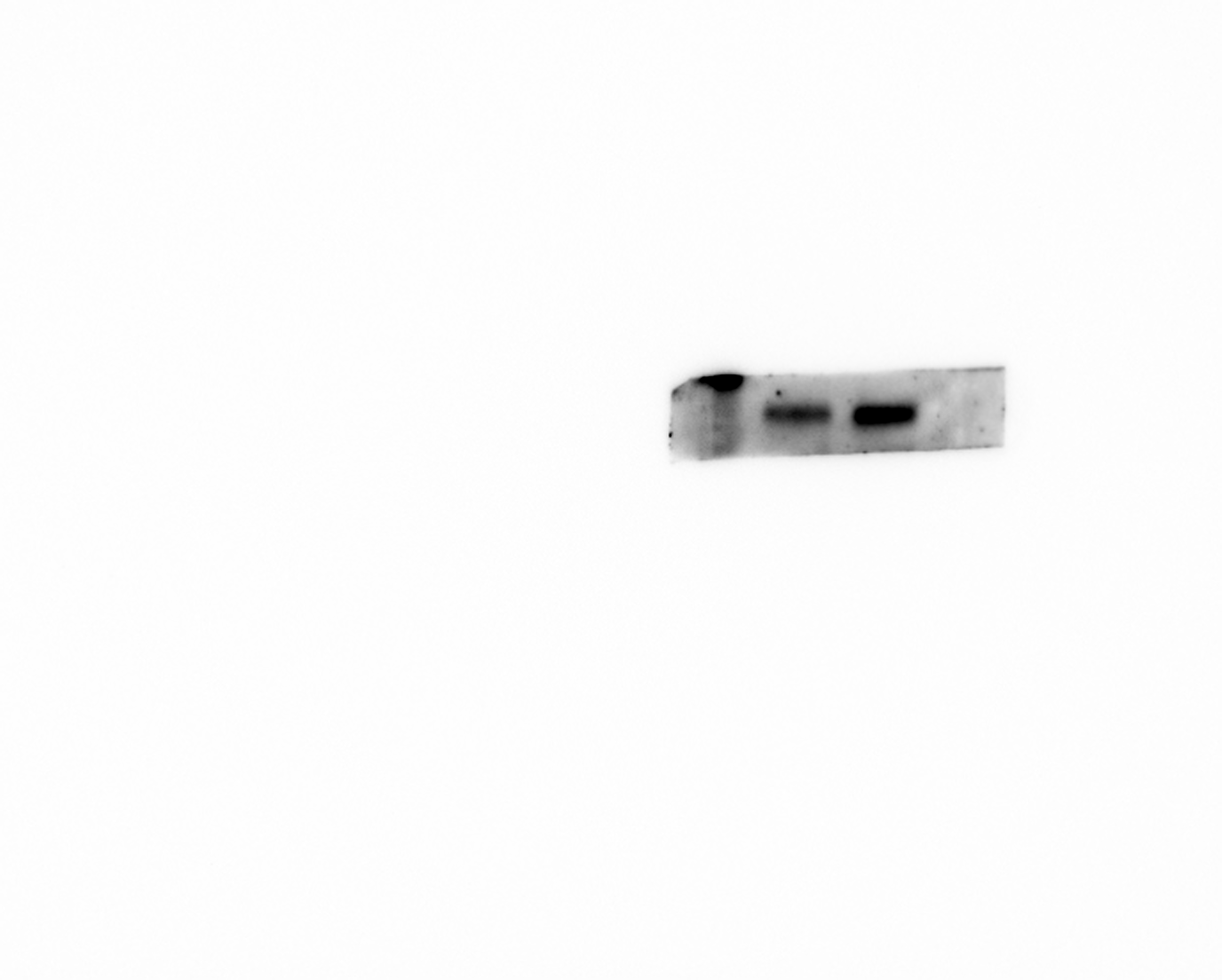

Supplement: Supplementary file 11 — Supplementary Material 11 [file 41598_2026_35675_MOESM11_ESM.tif]

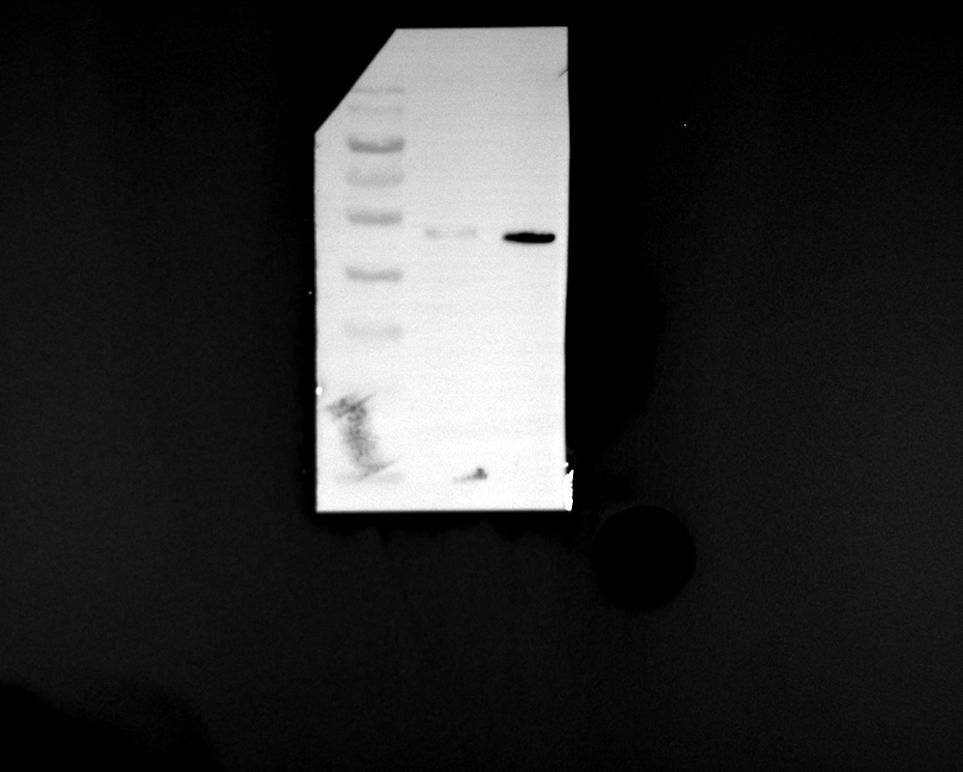

Supplement: Supplementary file 12 — Supplementary Material 12 [file 41598_2026_35675_MOESM12_ESM.tif]

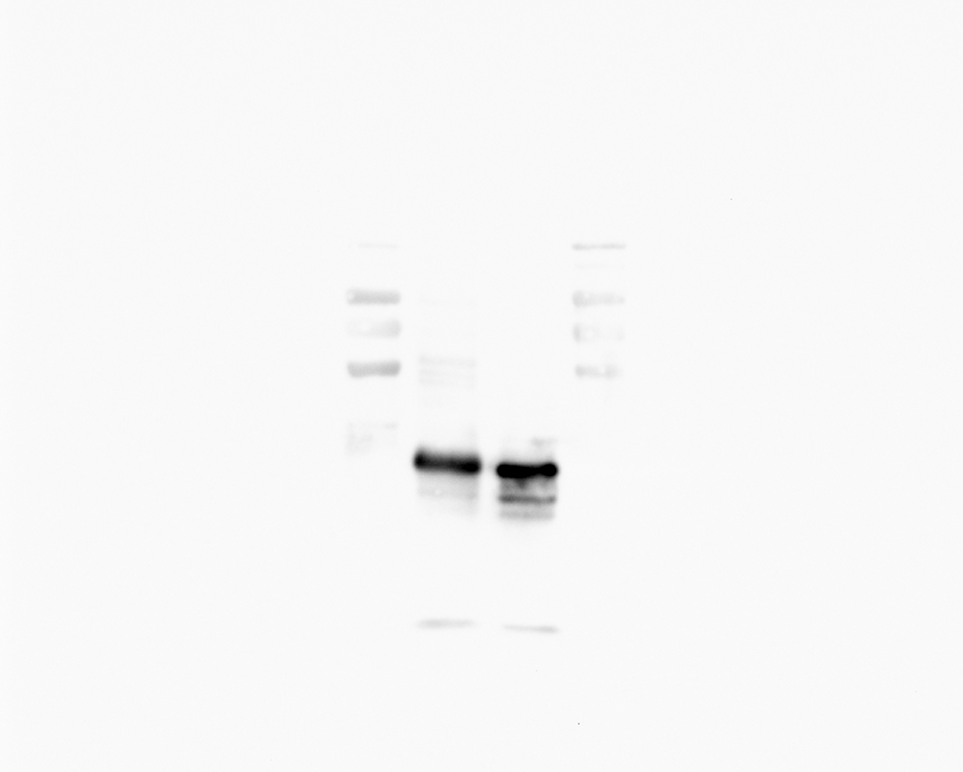

Supplement: Supplementary file 13 — Supplementary Material 13 [file 41598_2026_35675_MOESM13_ESM.tif]

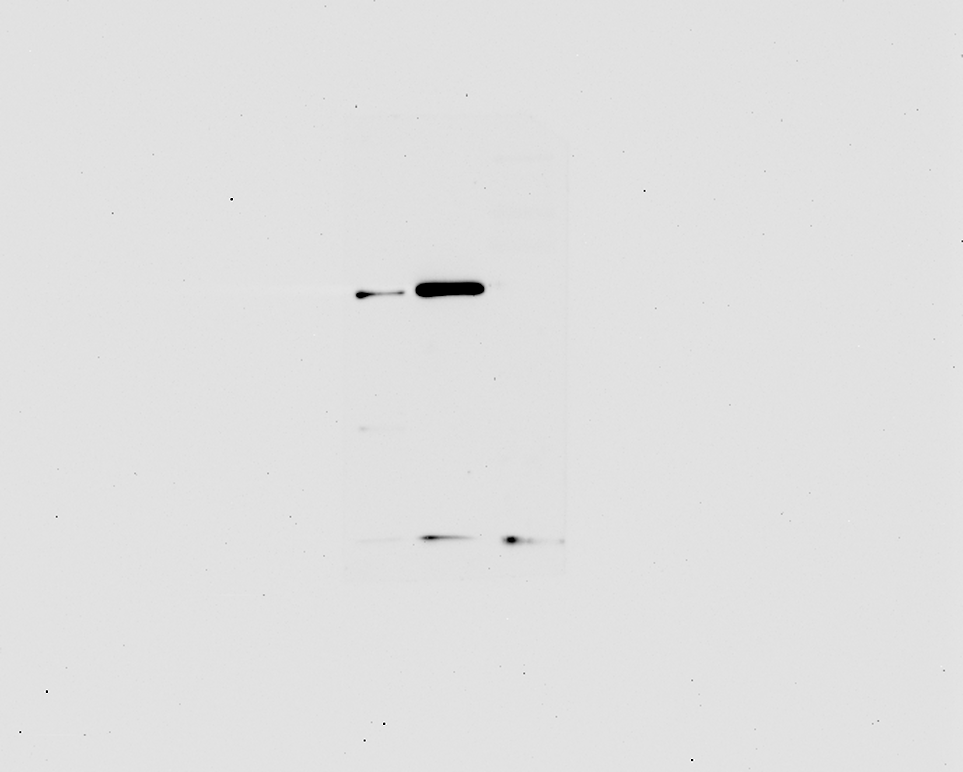

Supplement: Supplementary file 14 — Supplementary Material 14 [file 41598_2026_35675_MOESM14_ESM.tif]

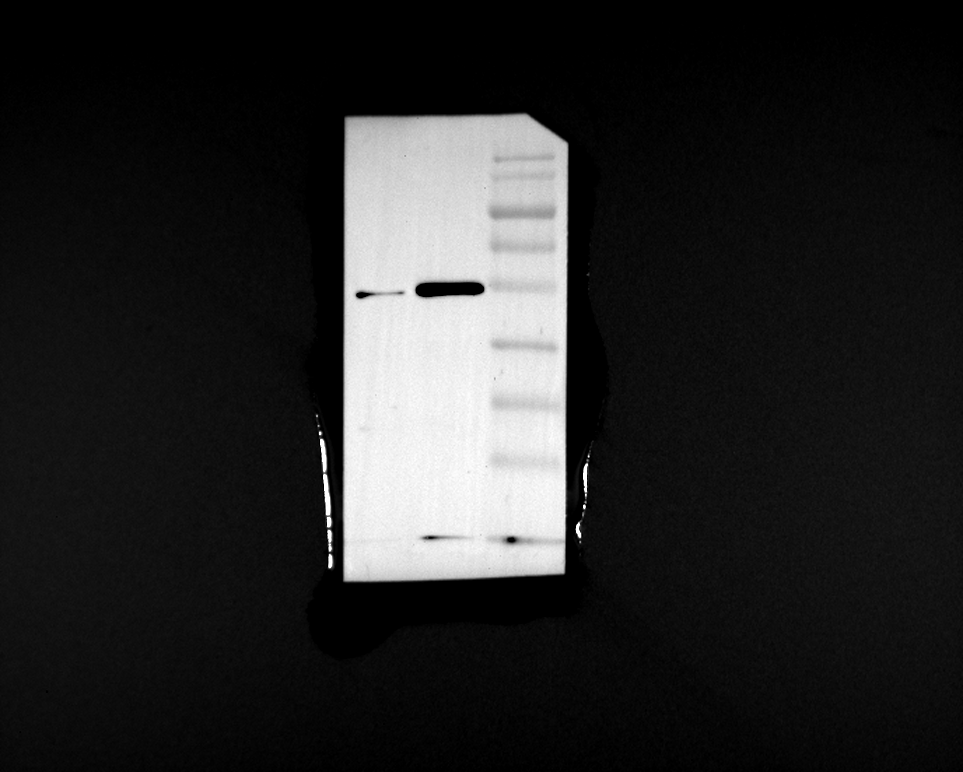

Supplement: Supplementary file 15 — Supplementary Material 15 [file 41598_2026_35675_MOESM15_ESM.tif]
